# ﻿Species diversity and systematic taxonomy of Sarcosomataceae (Ascomycota, Pezizales), with an emphasis on subtropical regions of China

**DOI:** 10.3897/mycokeys.121.155432

**Published:** 2025-08-21

**Authors:** Jin Rong Liu, Deng Li, Si Ang Chen, Yan Cheng Zhang, Guang Fu Mou, Yan Liu, Guang Rong Zhou, Zhou Rong Tan, Jian Hua Zhang

**Affiliations:** 1 Guangxi Key Laboratory of Plant Conservation and Restoration Ecology in Karst Terrai, Guangxi Institute of Botany, Guangxi Zhuang Autonomous Region and Chinese Academy of Sciences, Guilin 541006, Guangxi, China; 2 College of Life Science, Guangxi Normal University, Guilin 541006, Guangxi, China; 3 College of Life Sciences, Fujian Agriculture and Forestry University, Fuzhou, 350002, China; 4 College of Agriculture, Fujian Agriculture and Forestry University, Fuzhou 350002, China; 5 Managing Bureau of Damingshan National Nature Reserve, Wuming 530114, Guangxi, China; 6 Managing Bureau of Huaping National Nature Reserve, Guilin 531199, Guangxi, China; 7 Managing Bureau of Mao’ershan National Nature Reserve, Guilin 541316, Guangxi, China

**Keywords:** New taxa, phylogeny, *
Plectania
*, *
Pseudoplectania
*, subtropical fungus, *
Urnula
*

## Abstract

The aim of this study is to update the species diversity of Sarcosomataceae in subtropical China. Taxonomic and phylogenetic studies were carried out through ecological observation, morphological examination, and molecular phylogenetic analyses inferred from five molecular loci (ITS + nrLSU + *rpb1* + *rpb2* + *tef1-α*). By using Bayesian inference (BI) and maximum likelihood (ML) methods, six lignicolous species were identified, including four novel taxa: *Plectania damingshanensis***sp. nov.**, *Plectania huapingensis***sp. nov.**, *Pseudoplectania aureonigrescens***sp. nov.**, and *Urnula auricularioides***sp. nov.**; and two new distribution records: *Plectania lutea* and *Urnula ailaoshanensis*. Keys to species of *Plectania*, *Pseudoplectania*, and *Urnula* were provided.

## ﻿Introduction

Sarcosomataceae Kobayasi is regarded as an apothecial family of Pezizales within Ascomycota. This family has been studied for many decades, and its systematics could even date back to the 19^th^ century, spanning more than 170 years. Kobayasi (1937) originally proposed Sarcosomataceae (originally written as “Sarcosomaceae”) as an independent family based on its dark apothecia and highly gelatinized tissues, typified by the genus *Sarcosoma* Casp. However, based on the color of ascomata and the presence of subcystidia, Le Gal (1946) proposed a preliminary classification with an invalid family without diagnosis, Sarcoscyphaceae Le Gal ex Eckblad, further divided into two tribes: the bright-colored tribe Sarcoscypheae Le Gal and the brown-black tribe Urnuleae Le Gal. Subsequently, this single family was validated by Eckblad. Following the transfer of *Sarcosoma* into Urnuleae, the family Sarcosomataceae was temporarily considered a synonym (Eckblad 1968). Notably, taxa that primarily belonged to the tribe Urnuleae have now been classified under Sarcosomataceae.

In 1970, Korf proposed an ameliorative concept to fit the natural classification of these two families and divided Sarcosomataceae into two tribes: Galielleae Korf and Sarcosomatae Korf. With the restoration of Sarcosomataceae as an independent family, ten genera were successively recognized and classified into this family: *Chorioactis* Kupfer ex Eckblad, *Desmazierella* Libert, *Galiella* Nannf. & Korf, *Nannfeldtiella* Eckblad, *Neournula* Paden & Tylutki, *Plectania* Fuckel, *Pseudoplectania* Fuckel, *Sarcosoma*, *Urnula* Fr., and *Wolfina* Seaver ex Eckblad (Eckblad 1968; Korf 1970, 1972, 1973). Previous studies (Samuelson 1975; Samuelson et al. 1980; Brummelen 1981) have detailed the characteristics of the suboperculum and operculum in the asci of various members of Sarcosomataceae, including *Galiella*, *Pseudoplectania*, *Sarcosoma*, and *Urnula*. These studies indicated that these fungi exhibit the greatest morphological similarity to the non-eccentric type, particularly in their dehiscent structure, which features a thin-walled central cylinder measuring between 0.5 μm and 0.8 μm. Additionally, the apical annulus is typically thinner than the lateral wall, distinguishing these characteristics from those observed of Sarcoscyphaceae species. Furthermore, other research focusing on septal structures and spore ontogeny, utilizing numerical analysis methods and chemotaxonomic markers, has provided additional support for Korf’s perspective (Li and Kimbrough 1995, 1996; Köpcke et al. 2002).

With the rapid development of molecular techniques and their application in the taxonomy of macrofungi (Lücking et al. 2021), DNA sequence data have been widely used in systematic studies of Sarcosomataceae. Harrington et al. (1999) used 18S rDNA sequences to conduct an in-depth exploration of the intergeneric relationships within Discomycetes, including 10 genera of Sarcosomataceae and 11 genera of Sarcoscyphaceae. The results showed that Sarcosomataceae was not monophyletic. Subsequently, Pfister et al. (2008) carried out a phylogenetic study inferred from nrLSU, SSU, and *rpb2* sequence data, describing the new family Chorioactidaceae Pfister, with four genera transferred: *Chorioactis*, *Desmazierella*, *Neournula*, and *Wolfina*, while Sarcosomataceae was restricted to a strongly supported group with six sexual genera: *Donadinia*, *Galiella*, *Plectania*, *Pseudoplectania*, *Sarcosoma*, and *Urnula*. The abbreviations for the generic names referenced in this study are as follows: *D.* = *Donadinia*, *G.* = *Galiella*, *P.* = *Plectania*, *P..* = *Pseudoplectania*, *U.* = *Urnula*.

Carbone et al. (2013a) clarified the taxonomic framework of Sarcosomataceae, which supported the monophyly of these six genera, as shown in the phylogeny based on a broader sequence dataset. Nonetheless, the monotypic genus *Korfiella* D.C. Pant & V.P. Tewari, described from India (Pant and Tewari 1970), still lacks related molecular data. In addition, two asexual genera are currently accepted in Sarcosomataceae, viz. *Conoplea* Pers. and *Strumella* Fr., whose status in classification remains unclear and doubtful (Wijayawardene et al. 2017; Pfister and LoBuglio 2018). Previous phylogenetic studies (Harrington et al. 1999; Carbone et al. 2013a; Zeng et al. 2021) seemed to reveal the dispersed phylogenetic position of these asexual taxa within clades of other sexual genera.

Currently, species of Sarcosomataceae are accepted to accommodate lignicolous or terricolous ascomycetous fungi, mainly macroscopically distinguished by brown or black, discoid or cupulate to deep funnel-shaped, leathery or gelatinous, sessile or substipitate to stipitate apothecia (Carbone et al. 2013a; Zeng et al. 2021). In terms of microscopic morphology, Sarcosomataceae species present a definite arrangement of excipulum consisting of a gelatinous medullary excipulum and an ectal excipulum with one or two types of external hairs; a hymenium consisting of two types of paraphyses; suboperculate, unitunicate, inamyloid asci; and globose, ellipsoid, or allantoid ascospores with or without ornamentation and gelatinous sheath (Calonge et al. 2003; Carbone et al. 2014a, 2015a; Ekanayaka et al. 2018; Lu et al. 2023; Lin et al. 2024). Particularly, spore shapes and ornamentation, combined with molecular evidence, are recognized as the main characteristics for taxonomic treatment at the genus level (Carbone et al. 2013a), whereas a reliable criterion is lacking for distinguishing species (Korf 1972; Calonge and Mata 2002; Calonge et al. 2003; Carbone et al. 2013a; Wang et al. 2018; Lu et al. 2023).

The genus *Galiella* was proposed by Nannfeldt and Korf (Korf 1957a) with the typification of *Galiella rufa* (Schwein.) Nannf. & Korf. In Korf’s generic concept, all bulgarioid or *Sarcosoma*-like fleshy species with cyanophilous and warty ascospores were assigned to this genus. However, based solely on morphological examination, the concept of *Galiella* has changed intermittently. *Galiella* was regarded as a synonym of *Sarcosoma* by Boedijn (1959) and Le Gal (1958, 1960), while the independent status of *Galiella* was widely recognized by most authors (Korf 1972, 1973; Otani 1980; Moravec 1983; Cao et al. 1992; Zhuang and Wang 1998; Zhuang 2004; Carbone et al. 2015b; Popov and Carbone 2021). Furthermore, Samuelson et al. (1980) conducted an in-depth study on the apical characteristics of asci in *Galiella* and *Sarcosoma*, demonstrating that there were significant differences in ultrastructure and cell chemistry between them. By using the only *G. rufa* sequence data available for *Galiella*, Harrington et al. (1999) and Carbone et al. (2013a) initially confirmed the distinct position of this genus in Sarcosomataceae. However, as the type species *Trichaleurina polytricha* Rehm was re-examined, two subtropical or tropical *Galiella* species were subsequently transferred to the restored genus *Trichaleurina*, viz. *Galiella celebica* (Henn.) Nannf. and *Galiella javanica* (Rehm) Nannf. & Korf (Carbone et al. 2013b). At present, only *Galiella amurensis* (Lj.N. Vassiljeva) Raitv. and *G. rufa* have been confirmed through both morphological examination and molecular data (Köpcke et al. 2002; Carbone et al. 2013a; Popov and Carbone 2021). According to the recent concepts of Carbone et al. (2015b), the main distinguishing characteristics of *Galiella* species are as follows: gelatinous apothecia, warty or verrucose ascospores, and smooth external hairs.

At present, two *Galiella* species (*Galiella amurensis* and *Galiella sinensis* J.Z. Cao) are known from China (Zhuang 2004; Popov and Carbone 2021). The taxonomic status of *Galiella sinensis* is noteworthy, given that this species was considered a synonym of *Galiella celebica* (Henn.) Nannf. (as *Trichaleurina celebica* (Henn.) M. Carbone, Agnello & P. Alvarado) by Zhuang and Wang (1998). However, Carbone et al. (2013b) pointed out that the type specimen (HMAS 29566) of *G. sinensis* was more closely related to a true *Galiella* species based on the smooth hairs they observed.

The genus *Plectania* was established by Fuckel (1870) and was typified by *Plectania melastoma* (Sowerby) Fuckel (Sowerby 1799). Moreover, this genus was divided into five sections based only on spore shapes and ornamentation (Korf 1957a; Paden JW 1983; Carbone et al. 2012): Plectania sect. Plectania Fuckel, Plectania sect. Plicosporae Korf, Plectania sect. Curvatisporae Korf, Plectania sect. Sphaerosporae Paden (= *Pseudoplectania*), and Plectania sect. Donadinia (Bellem. & Mel.-Howell) M. Carbone & Agnello (= *Donadinia*). As shown by the phylogenetic analysis of Carbone et al. (2013a), the *Plectania* clade is monophyletic based on limited sequence data sets and molecular loci. However, the work of Zeng et al. (2021) and Hyde et al. (2024), which adopted more sequence data, showed that *Plectania* species did not cluster together and instead formed a paraphyletic group closely related to *Galiella*. In the current sense, *Plectania* is characterized by the following features: the ascocarp is discoid to deeply cupulate, sessile to stipitate, with a dark brown to nearly black external surface covered with dark hairs; the ectal excipulum is composed of angular cells, and the medullary excipulum consists of a *textura intricata* and is often gelatinous; the mature ascospores are elliptical, smooth or ornamented (transversally striated or verrucose), with or without a gelatinous sheath (Carbone et al. 2013a, 2015a; Ekanayaka et al. 2018).

Based on a study of Chinese specimens of Sarcosomataceae, Zhuang (2004) catalogued and described six species of *Plectania* in China: *P. campylospora* (Berk.) Nannf. (a synonym of *Urnula campylospora* (Berk.) Cooke), *P. melastoma* (Sowerby) Fuckel, *P. nannfeldtii* Korf (a synonym of *Donadinia nigrella* (Seaver) M. Carbone, Agnello & P. Alvarado), *P. platensis* (Speg.) Rifai, *P. rhytidia* (Berk.) Nannf. & Korf, and *P. yunnanensis* W.Y. Zhuang (Zhuang and Wang 1998; Zhuang 2004). In recent years, two novel species have been reported from subtropical China: *Plectania lutea* T. Bau & G.F. Mou (Mou and Bau 2021) and *P. sichuanensis* M. Zeng, Q. Zhao & K.D. Hyde (Zeng et al. 2021).

The genus *Pseudoplectania* was initially erected by Fuckel in 1870. The typical representative of this genus, *Ps. nigrella* (Pers.) Fuckel, had already been documented by Persoon (1801) and was formally designated as the type species of the genus by Korf (1972). For a long time, *Pseudoplectania* was considered an independent genus by Seaver (1928, 1942), Nannfeldt (1949), Le Gal (1953), Sanwal (1953), Kreisel (1962), and Berthet (1964), standing out from other sarcosomataceous fungi by its spherical spores. Nevertheless, Korf (1982) and Paden (1983) argued that this genus should be treated as a synonym of *Plectania*, amending it due to its *Conoplea* conidial state and the rounded spores of *Plectania* when young.

In 2013, the work of Carbone et al. (2013a) inferred that the genus *Pseudoplectania* is a monophyletic clade based on phylogenetic analysis. To date, species of *Pseudoplectania* are distinguished by tiny to medium-large ascomata, sessile, shortly stipitate to distinctly stipitate, with a dark brown to black external surface often bearing basal tomentum; an ectal excipulum with a *textura subglobulosa* to *prismatica angularis*; one or two types of straight, curved, flexuous, or coiled external hairs; and, in particular, smooth, thick-walled, globose to subglobose mature spores with or without a gelatinous sheath, distinguishing them from other genera in Sarcosomataceae (Carbone et al. 2013a, 2014a; Lin et al. 2024). Only four species of *Pseudoplectania* have been reported in China, namely *Pseudoplectania globospora* M. Zeng & Q. Zhao, *Ps. mystica* Jia Y. Lin, Ling Han Guo & Kun L. Yang, *Ps. nigrella*, and *P. sinica* Qiao Zhang & Jie Zhang (Zhuang 2018; Zhang and Zhang 2020; Lin et al. 2024; Zeng et al. 2024).

The genus *Urnula* was established by Fries, with *Urnula craterium* (Schwein.) Fr. designated as the type species (Fries 1849, 1851). However, some *Urnula* species were previously placed in *Plectania* and *Gloeocalyx* Massee (a later synonym of *Urnula*) due to the significant morphological differences from the type species *U. craterium*, e.g., *U. campylospora* (Korf 1957b; Rifai 1968; Carbone and Agnello 2013). Subsequently, Carbone et al. (2013a) conducted an in-depth phylogenetic study, revealing a monophyletic group of *Urnula* species with strong support. Several species originally placed in *Urnula*, such as *Urnula megalocrater* Malençon & Le Gal, *U. lusitanica* Torrend & Boud., and *U. platensis* Speg., were transferred to other genera. Currently, this genus is recognized by its dark, bowl- to funnel-shaped ascomata; stipitate or sessile fruitbodies; absence of crystals in the hymenium; one or two types of external hairs; gelatinous medullary excipulum consisting of a *textura intricata*; ectal excipulum with *textura subglobulosa* to *angularis*; and especially by its smooth, elliptical to allantoid mature ascospores, distinguishing it from other genera in Sarcosomataceae (Carbone et al. 2013a; Wang et al. 2018; Lu et al. 2023). These exceedingly similar morphological features provide little interspecific heterogeneity, making *Urnula* species difficult to distinguish (Wang et al. 2018; Lu et al. 2023).

There are relatively few previous studies or reports on the classification and diversity of *Urnula* species in China. Only five published species have been reported: *Urnula ailaoshanensis* J.R. Lu, Ying Zhang & Q. Zhao, *U. campylospora* (Berk.) Cooke, *U. craterium* (Schwein.) Fr., *U. subcrateria* J.R. Lu, F.M. Yu & Q. Zhao, and *U. versiformis* Y.Z. Wang & Cheng L. Huang (Nannfeldt 1949; Zhuang and Wang 1998; Zhuang 2004; Wang and Huang 2015; Lu et al. 2023).

In this study, twelve specimens collected from subtropical China were newly examined and sequenced. Through field sampling, microscopic studies, ultrastructural observation, and phylogenetic analysis based on five molecular loci (ITS + nrLSU + *rpb1* + *rpb2* + *tef1-α*), four new species were confirmed: *Plectania damingshanensis*, *P. huapingensis*, *Pseudoplectania aureonigrescens*, and *Urnula auricularioides*. Two new distribution records are also reported: *P. lutea* and *U. ailaoshanensis*. Keys to the accepted species of *Pseudoplectania*, *Urnula*, and Chinese species of *Plectania* are also provided.

## ﻿Materials and methods

### ﻿Sampling, morphological observations, and descriptions

Twelve specimens for this study were collected from Guangxi Zhuang Autonomous Region (abbreviated as Guangxi below) and Fujian Province in China and deposited in the Herbarium of Guangxi Institute of Botany (IBK, Guangxi, China). Macro-morphological descriptions and measurements were based on fresh specimens. Micro-morphological data were obtained from dried specimens and observed under an optical microscope and scanning electron microscope. Color codes followed the standards established by Kornerup and Wanscher (1978). Microscopic features were studied on air-dried samples after hand sectioning and rehydrating in distilled water and, if necessary, mounting in Congo red under suitable light conditions. The iodine reaction of asci and ascospores was tested using Melzer’s reagent. For optimal microscopic observation, this study utilized a Nikon E80i microscope (Japan) to examine the sections at magnifications of ×100, ×200, ×400, ×600, and ×1000.

For electron microscopy, the ultrastructure of ascospores was observed using a Zeiss EVO18 scanning electron microscope (Carl Zeiss IMT (Shanghai) Co., Ltd., Shanghai, China). The ascospore dimensions are described as follows, excluding ornamentation: [*a*/*b*/*c*] length × width, and the factor Q, which is the ratio of spore length to width. The measured value from *a* ascospores of *b* ascomata in *c* specimens is shown as (*d*) *e*–*f* (*g*), where *e–f* represents at least 90% of the values, *d* is the minimum, and *g* is the maximum value. Thirty or more mature and rehydrated ascospores were measured. In describing the morphological characteristics, terminology follows Carbone et al. (2013a, 2014a), Zeng et al. (2021), Lu et al. (2023), Lin et al. (2024), and Zeng et al. (2024).

### ﻿DNA extraction and sequence amplification

DNA was extracted from dried specimens using the NuClean Plant Genomic DNA Kit (CWBIO). The primer pairs used included ITS4 and ITS5 for the internal transcribed spacer region (ITS) (White et al. 1990); LR0R and LR5 for the large subunit nuclear ribosomal RNA (nrLSU) (Vilgalys and Hester 1990); RPB1-Af and RPB1-Cf for the first largest subunit of RNA polymerase I gene (*rpb1*) (Matheny et al. 2002); fRPB2-5F and fRPB2-7.1R for the second largest subunit of RNA polymerase II gene (*rpb2*) (Matheny 2005); and EF1-983F and EF1-1567R for the translation elongation factor 1-α gene (*tef1-α*) (Rehner and Buckley 2005).

Polymerase chain reaction (PCR) was performed in a total volume of 30 μL, consisting of 15 μL of 2× Es Taq MasterMix, 9 μL of ddH_2_O, 1.5 μL of each forward and reverse primer, and 3 μL of DNA template. The PCR procedure for ITS and nrLSU was as follows: initial denaturation at 94 °C for 4 min; followed by 35 cycles at 94 °C for 40 s, 52 °C for 40 s, and 72 °C for 1 min; with a final extension at 72 °C for 10 min. The PCR procedure for *rpb1*, *rpb2*, and *tef1-α* was as follows: initial denaturation at 94 °C for 2 min, followed by denaturation at 94 °C for 40 s, annealing at 60 °C for 40 s with an increment of 1 °C per cycle, and extension at 72 °C for 2 min. This step was repeated 8 times. Then, denaturation at 94 °C for 45 s, annealing at 53 °C for 1.5 min, and extension at 72 °C for 2 min, repeated 36 times. Finally, a final extension was performed at 72 °C for 10 min. Successful PCR products were sent to Sangon Biotech (Shanghai) Co., Ltd., for sequencing. Sequence results were checked using Chromas v2.6.6 (https://technelysium.com.au/wp/chromas), and sequence data from the specimens were submitted to GenBank (https://www.ncbi.nlm.nih.gov/genbank).

### ﻿Data analysis

Sequences of Sarcosomataceae downloaded from GenBank mainly referred to Carbone et al. (2013c, 2014a), Zeng et al. (2021), Lu et al. (2023), and Lin et al. (2024). Additional unpublished sequences were also downloaded from GenBank (https://www.ncbi.nlm.nih.gov/genbank). Detailed information on the sequences included is shown in Table 1. A total of 128 ITS, 111 nrLSU, 11 *rpb1*, 17 *rpb2*, and 25 *tef1-α* sequences from Sarcosomataceae were used for molecular phylogenetic analysis. Sequences were aligned using Clustal W in BioEdit v7.1.3 with default parameters (Hall 1999). This study selected five species from two families, Chorioactidaceae Pfister and Pyronemataceae Corda, as outgroups of Sarcosomataceae (Perry et al. 2007; Pfister et al. 2008). Alignments were refined in MEGA 11 (Tamura et al. 2021). Individual alignments were concatenated for ITS + nrLSU + *rpb1* + *rpb2* + *tef1-α* using SequenceMatrix (Vaidya et al. 2011). The concatenated dataset was submitted to TreeBASE (ID: 32052).

**Table 1.** Taxa, sequences, and collections analyzed in this study. Collections in bold are newly sequenced in this study. The letters HT, ET, IT, and NT after the sample ID/voucher indicate holotype, epitype, isotype, and neotype, respectively. NA indicates sequences that are not available.

Specie	Sample ID/Voucher	GenBank accession					Reference
		ITS	nrLSU	rpb1	rpb2	tef1-α	
*Chorioactis geaster*	DHP 02.497	NA	KC012672	NA	NA	KC109211	Hansen et al. (2013)
*Conoplea fusca*	CBS: 113475	EU552114	NA	NA	NA	NA	Carbone et al. (2013a)
*C. fusca*	CHTAR77	NA	GU048612	NA	NA	NA	Carbone et al. (2013a)
*C. globosa*	CBS:233.91	AF485076	NA	NA	NA	NA	Köpcke et al. (2002)
*C. globosa*	CBS:438.51	AF485069	NA	NA	NA	NA	Köpcke et al. (2002)
*Conoplea* sp.	JCM12000	JQ972061	NA	NA	NA	NA	NCBI
*Donadinia echinacea*	HKAS 107659^HT^	MW077543	MW079923	MW084979	NA	MW094269	Zeng et al. (2021)
*D. echinacea*	HKAS 107660	MW077544	MW079924	NA	MW085092	NA	Zeng et al. (2021)
*D. echinacea*	HKAS 107661	MW077545	MW079925	NA	NA	NA	Zeng et al. (2021)
*D. echinacea*	HKAS 107662	MW077546	MW079926	NA	NA	MW094270	Zeng et al. (2021)
*D. helvelloides*	LY PB 940^IT^	JX669834	JX669872	NA	NA	NA	Carbone et al. (2013a)
*D. helvelloides*	SMPM206	KP794618	KP794617	NA	NA	NA	NCBI
*D. helvelloides*	MCVE 28377	KP204907	KP204914	NA	NA	NA	Carbone et al. (2014b)
*D. lusitanica*	MCVE 28378	KP204906	KP204913	NA	NA	NA	Carbone et al. (2014b)
*D. lusitanica*	TUR-A 195791	JX669811	JX669847	NA	NA	NA	Carbone et al. (2013a)
*D. lusitanica*	TUR-A 195792	JX669810	JX669846	NA	NA	NA	Carbone et al. (2013a)
*D. nigrella*	TUR-A 195793	JX669836	JX669874	NA	NA	NA	Carbone et al. (2013a)
*D. nigrella*	WTU-F-017148	KP204912	KP204919	NA	NA	NA	Carbone et al. (2014b)
*D. nigrella*	WTU-F-017150	KP204911	KP204918	NA	NA	NA	Carbone et al. (2014b)
*D. nigrella*	CBS:694.71	MH860304	NA	NA	NA	NA	NCBI
*D. nigrella*	KH-97-16 (FH)	NA	AY945853	JX943641	DQ017592	KC109214	Hansen et al. (2013)
*D. seaveri*	CUP-Whetz.B.F. 0188^HT^	KC249999	NA	NA	NA	NA	Carbone et al. (2014b)
*D. seaveri*	FH 00458441	KY794720	KY794715	NA	NA	NA	Harrington et al. (1999)
*D. seaveri*	FH 01142449^IT^	KY794717	KY794712	NA	NA	NA	Carbone et al. (2013a)
*D. sibirica*	MCVE 28374^HT^	KP204910	KP204917	NA	NA	NA	Carbone et al. (2014b)
*D. sibirica*	MCVE 28375	KP204908	KP204915	NA	NA	NA	Carbone et al. (2014b)
*Donadinia* sp.	mh 699 (FH)	NA	DQ220329	NA	DQ017593	NA	Perry et al. (2007)
*Galiella amurensis*	LE 236216	MW879699	MW879115	NA	NA	NA	Popov and Carbone (2021)
*G. amurensis*	LE 323821	MW879700	MW879116	NA	NA	NA	Popov and Carbone (2021)
*G. amurensis*	LE 323822^ET^	MW879701	MW879117	NA	NA	NA	Popov and Carbone (2021)
*G. rufa*	CBS:135.92	AF485070	FJ176869	NA	NA	NA	Köpcke et al. (2002)
*G. rufa*	CBS:762.85	AF485072	KC012674	NA	NA	NA	Pfister et al. (2018)
*G. rufa*	DHP 05.600 (FH)	NA	KC012674	JX943642	NA	KC109213	Hansen et al. (2013)
*G. rufa*	AFTOL-ID 1297	NA	FJ176869	NA	FJ238352	FJ238401	Schoch et al. (2009)
*Neournula pouchetii*	TUR-A 195798	JX669837	JX669875	NA	NA	NA	Carbone et al. (2013a)
*N. pouchetii*	MO-205345	KT968605	KT968655	NA	NA	NA	NCBI
***Plectania damingshanensis***	**MES2024040606**	**PQ691392**	**PQ682446**	**PV247119**	**NA**	**PV295999**	**This study**
***P. damingshanensis***	**DMS2024022001^HT^**	**PP864720**	**PP859016**	**PV247118**	**NA**	NA	**This study**
***P. damingshanensis***	**DMS2024022002**	**PP864721**	**PP859017**	**NA**	**NA**	**PV295998**	**This study**
***P. damingshanensis***	**DMS2024022003**	**PP864722**	**PP859018**	**NA**	**NA**	**NA**	**This study**
*P. fisherae*	MST-F22868	PQ066526	PQ060437	NA	PQ067358	NA	Yu et al. (2024)
*P. harnischii*	TUR-A 195785	NR_174881	NG_079625	NA	NA	NA	Carbone et al. (2021)
*P. harnischii*	WTU-F-68864	MZ713186	MZ713201	NA	NA	NA	Carbone et al. (2021)
***P. huapingensis***	**HP2024071903 ^HT^**	**PQ691391**	**PQ682445**	**NA**	**PV296007**	**PV296000**	**This study**
***P. huapingensis***	**HP2024072014**	**PQ691390**	**PQ682444**	**NA**	**NA**	**NA**	**This study**
*P. lutea*	HMJAU 57091^HT^	NR_189838	NG_242497	NA	NA	NA	Mou and Bau (2021)
*P. lutea*	IBKM01	MW794211	MW794213	NA	NA	NA	Mou and Bau (2021)
***P. lutea***	**MES2024033005**	**PP859394**	**PP981371**	**PV247120**	**PV296006**	**PV296001**	**This study**
*P. megalocrater*	TUR-A 195803	JX669809	JX669845	NA	NA	NA	Carbone et al. (2013a)
P. cf. melastoma	TUR-A 195785	JX669804	JX669840	NA	NA	NA	Carbone et al. (2013a)
*P. melastoma*	TUR-A 195783	JX669805	JX669841	NA	NA	NA	Carbone et al. (2013a)
*P. melastoma*	TUR-A 195784	JX669814	JX669850	NA	NA	NA	Carbone et al. (2013a)
*P. melastoma*	MH679	NA	NA	NA	MN103434	MN103422	Pfister et al. (2019)
*P. milleri*	OSC 104436	EU652354	EU652389	NA	NA	NA	NCBI
*P. milleri*	OSC 73165	EU652355	EU652390	NA	NA	NA	NCBI
*P. milleri*	TUR-A 190823	JX669812	JX669848	NA	NA	NA	Carbone et al. (2013a)
*P. rhytidia*	PDD 90028	JX669832	JX669871	NA	NA	NA	Carbone et al. (2013a)
*P. rhytidia*	TUR-A 195786	JX669813	JX669849	NA	NA	NA	Carbone et al. (2013a)
*P. rhytidia*	TUR-A 195787	JX669815	JX669851	NA	NA	NA	Carbone et al. (2013a)
*P. rhytidia*	TUR-A 195788	JX669816	JX669852	NA	NA	NA	Carbone et al. (2013a)
*P. sichuanensis*	HKAS 107664^HT^	MW077547	MW079927	NA	MW085093	MW094271	Zeng et al. (2021)
*P. submilleri*	HKAS 129665^HT^	OR500919	OR500933	NA	NA	NA	Hyde et al. (2024)
*P. submilleri*	HKAS 129666	OR500920	OR500934	NA	NA	NA	Hyde et al. (2024)
*Plectania* sp.	Mumblo 2007090-01	JX310423	JX287505	NA	NA	NA	NCBI
*Plectania* sp.	PA39	MG543948	NA	NA	NA	NA	NCBI
*P. zugazae*	AVM1467^HT^	JX669817	JX669854	NA	NA	NA	Pfister et al. (2019)
*P. zugazae*	AVM2086	JX669818	JX669855	NA	NA	NA	Pfister et al. (2019)
*P. zugazae*	TUR-A 199785	KM610322	KM610324	NA	NA	NA	Carbone et al. (2015)
*Pseudoplectania affinis*	PDD 81842^HT^	JX669826	JX669865	NA	NA	NA	Carbone et al. (2014a)
*Ps. affinis*	RBG 7287	ON989641	NA	NA	OP066979	OP066929	NCBI
*Ps. africana*	TUR-A 216892	PP590544	PP587623	NA	NA	NA	Pfister et al. (2019)
*Ps. africana*	ZE59/18^HT^	MT496892	MT496884	NA	OR961521	OR961523	Pfister et al. (2019)
*Ps. africana*	TUR-A 216593	OR940185	OR939326	NA	OR961522	OR961524	Pfister et al. (2019)
***Ps. aureonigrescens***	**CSA-797**	**PQ863322**	**PQ863491**	**NA**	**NA**	**PV296004**	**This study**
***Ps. aureonigrescens***	**CSA-798^HT^**	**PQ895821**	**PQ863683**	**PV247121**	**NA**	**PV296005**	**This study**
*P.. episphagnum*	TUR 064171	KF305712	NA	NA	NA	NA	Carbone et al. (2013a)
*P.. episphagnum*	TUR 064173	KF305711	KF305724	NA	NA	NA	Carbone et al. (2013a)
*Ps. ericae*	MCVE 27581	KF305721	KF305731	NA	NA	NA	Carbone et al. (2014a)
*Ps. ericae*	TUR-A 195789	JX669822	JX669862	NA	NA	NA	Carbone et al. (2013a)
*Ps. ericae*	MPU: JCD 82-775	MT273641	NA	NA	NA	MT274711	Pfister et al. (2019)
*Ps. globospora*	HKAS 127988^HT^	PP357153	OR879981	NA	NA	NA	Zeng et al. (2024)
*Ps. globospora*	HKAS 127989	PP357154	OR879982	NA	NA	NA	Zeng et al. (2024)
*Ps. lignicola*	HR89756	MT496886	MT496882	NA	NA	NA	Pfister et al. (2019)
*Ps. lignicola*	SAV 105/17	MT496881	MT496883	NA	NA	NA	Pfister et al. (2019)
*Ps. melaena*	MCVE 27433	JX669806	JX669842	NA	NA	NA	Carbone et al. (2015)
*Ps. melaena*	MCVE 27579	KF305717	KF305728	NA	NA	NA	Carbone et al. (2015)
*Ps. melaena*	NY 54130	KF305718	KF305730	NA	NA	NA	Carbone et al. (2015)
*Ps. mystica*	HKAS133073^HT^	PP422101	PP422115	NA	NA	NA	Lin et al. (2024)
*Ps. mystica*	HKAS133074	PP422102	PP422114	NA	NA	NA	Lin et al. (2024)
*Ps. nigrella*	KL BK-4914^NT^	JX669807	JX669843	NA	NA	NA	Carbone et al. (2013a)
*Ps. nigrella*	MCVE 27396	KF305715	KF305725	NA	NA	NA	Carbone et al. (2015)
*Ps. nigrella*	TUR 169888	JX669821	JX669859	NA	NA	NA	Carbone et al. (2013a)
*Ps. nigrella*	FH 00822741	NA	NA	NA	MN103433	MN103421	Pfister et al. (2019)
*P.. sinica*	CGMCC 3.19892	NR_171866	NA	NA	NA	NA	NCBI
*P.. sinica*	CGMCC 3.19892	MN814441	NA	NA	NA	NA	Zhang and Zhang (2020)
*Pseudoplectania* sp.	3-1-7-2-4-1	KX065280	NA	NA	NA	NA	Li et al. (2016)
*Pseudoplectania* sp.	DO87	KP050642	NA	NA	NA	NA	NCBI
*Pseudoplectania* sp.	ER1858	MZ091916	MZ019023	NA	NA	NA	Healy et al. (2022)
*Pseudoplectania* sp.	QU0896	MZ091877	MZ019019	NA	NA	NA	Healy et al. (2022)
*P.. tasmanica*	MCVE 27584	KF305723	KF305733	NA	NA	NA	Carbone et al. (2014a)
*P.. tasmanica*	MCVE 27583^HT^	KF305722	KF305732	NA	NA	NA	Carbone et al. (2014a)
*Pseudosarcosoma latahensis*	TUR-A 195801	JX669819	JX669856	NA	NA	NA	Carbone et al. (2013a)
*Sarcosoma globosum*	KH.07.04 (S)	FJ499393	NA	JX943640	JX943753	KC109215	NCBI
*S. globosum*	LE-BIN 3794	KY344789	NA	NA	NA	NA	NCBI
*S. globosum*	AJ1324	OR529395	NA	NA	NA	OR543971	NCBI
*Trichaleurina tenuispora*	TUR-A 195800	JX669839	JX669876	NA	NA	NA	Carbone et al. (2013a)
*T. javanica*	TUR-A 195799	JX669838	JX669861	NA	NA	NA	Carbone et al. (2013a)
*T. javanica*	HKAS 88981	MG871291	MG871326	NA	MG980716	MG980693	NCBI
*Urnula ailaoshanensis*	HKAS129212^HT^	OQ941816	OQ941895	NA	NA	NA	Lu et al. (2023)
*U. ailaoshanensis*	HKAS129213	OQ941817	OQ941896	NA	NA	NA	Lu et al. (2023)
***U. ailaoshanensis***	**MES2023081104**	**PQ187434**	**PQ187435**	**PV247122**	**NA**	**NA**	**This study**
***U. auricularioides***	**CSA-455^HT^**	**PQ489315**	**PQ187431**	**PV247123**	**PV296008**	**PV296003**	**This study**
***U. auricularioides***	**CSA-454**	**PQ489472**	**PQ187433**	**NA**	**NA**	**PV296002**	**This study**
*U. campylospora*	PDD 83522	JX669830	JX669869	NA	NA	NA	Carbone and Agnello (2013)
*U. campylospora*	PDD 88805	JX669831	JX669870	NA	NA	NA	Carbone and Agnello (2013)
*U. craterium*	30.15.291.11	KF311230	KF305734	NA	NA	NA	Carbone et al. (2014a)
*U. craterium*	TUR-A 195794	JX669820	JX669857	NA	NA	NA	Carbone et al. (2013a)
*U. craterium*	DHP 04-511 (FH)	NA	AY945851	JX943639	DQ017595	KC109216	Hansen et al. (2013)
*U. hiemalis*	TUR 136909	JX669827	JX669867	NA	NA	NA	Carbone et al. (2013a)
*U. hiemalis*	TUR 196076	JX669828	JX669868	NA	NA	NA	Carbone et al. (2013a)
*U. hiemalis*	TUR-A 195795	JX669835	JX669873	NA	NA	NA	Carbone et al. (2013a)
*U. himalayana*	CAL 1673^HT^	NR_159073	NG_064532	NA	NA	NA	Wang et al. (2018)
*U. himalayana*	CAL 1674	MH179123	MH179124	NA	NA	NA	Wang et al. (2018)
*U. mediterranea*	MCVE 28636	KU933925	KU933926	NA	NA	NA	Marziani et al. (2016)
*U. mediterranea*	TUR-A 195796	JX669808	JX669844	NA	NA	NA	Carbone et al. (2013a)
*U. mediterranea*	TUR-A 195797	JX669824	JX669864	NA	NA	NA	Carbone et al. (2013a)
*U. padeniana*	WTU-F-33051^HT^	JX669825	JX669866	NA	NA	NA	Carbone et al. (2013a)
*U. subcrateria*	HKAS129214^HT^	OQ944116	OQ941897	NA	NA	NA	Lu et al. (2023)
*U. subcrateria*	HKAS129215	OQ944117	OQ941898	NA	NA	NA	Lu et al. (2023)
***Urnula* sp.**	**YC2023090928**	**PQ550998**	**PQ187037**	**NA**	**NA**	**NA**	**This study**
*Urnula* sp.	EN-22	KT358892	NA	NA	NA	NA	Healy et al. (2022)
*Urnula* sp.	GL1-D-D1	KX100405	NA	NA	NA	NA	Strobel et al. (2017)
*Urnula* sp.	PDD 81259	JX669829	NA	NA	NA	NA	Carbone and Agnello (2013)
*Urnula* sp.	TNM F15544	KJ577537	NA	NA	NA	NA	Wang and Huang (2015)
*U. versiformis*	TNM F11312	KJ577534	NA	NA	NA	NA	Wang and Huang (2015)
*U. versiformis*	TNM F11317^HT^	KJ577535	NA	NA	NA	NA	Wang and Huang (2015)
*U. versiformis*	TNM F13875	KJ577536	NA	NA	NA	NA	Wang and Huang (2015)

The optimal evolutionary model for the matrix was selected using MrModeltest v2.3 based on the Akaike Information Criterion (AIC) (Nylander et al. 2004). Maximum likelihood (ML) and Bayesian inference (BI) were both used for phylogenetic reconstruction. ML analysis was performed in RAxMLGUI v1.5b1 (Stamatakis et al. 2005) using the bootstrap method with 1,000 replicates and the rapid bootstrapping algorithm. BI analysis was conducted using MrBayes v3.2.7 (Ronquist and Huelsenbeck 2003) with Markov chain Monte Carlo (MCMC) simulation set at 5,000,000 generations, sampling trees and parameters every 1,000^th^ generation (two parallel runs; burn-in = 25%). Bayesian posterior probability (BPP) was used to assess branch support. Significance thresholds were defined as follows: MLB ≥ 50% and BPP ≥ 0.90 for well-supported branches; either MLB < 50% or BPP < 0.90 for moderate support; and MLB < 50% and BPP < 0.90 for unsupported branches. FigTree v1.4.4 was used to visualize the phylogram (Rambaut 2018).

## ﻿Results

### ﻿Phylogenetic analyses

**Figure 1.**
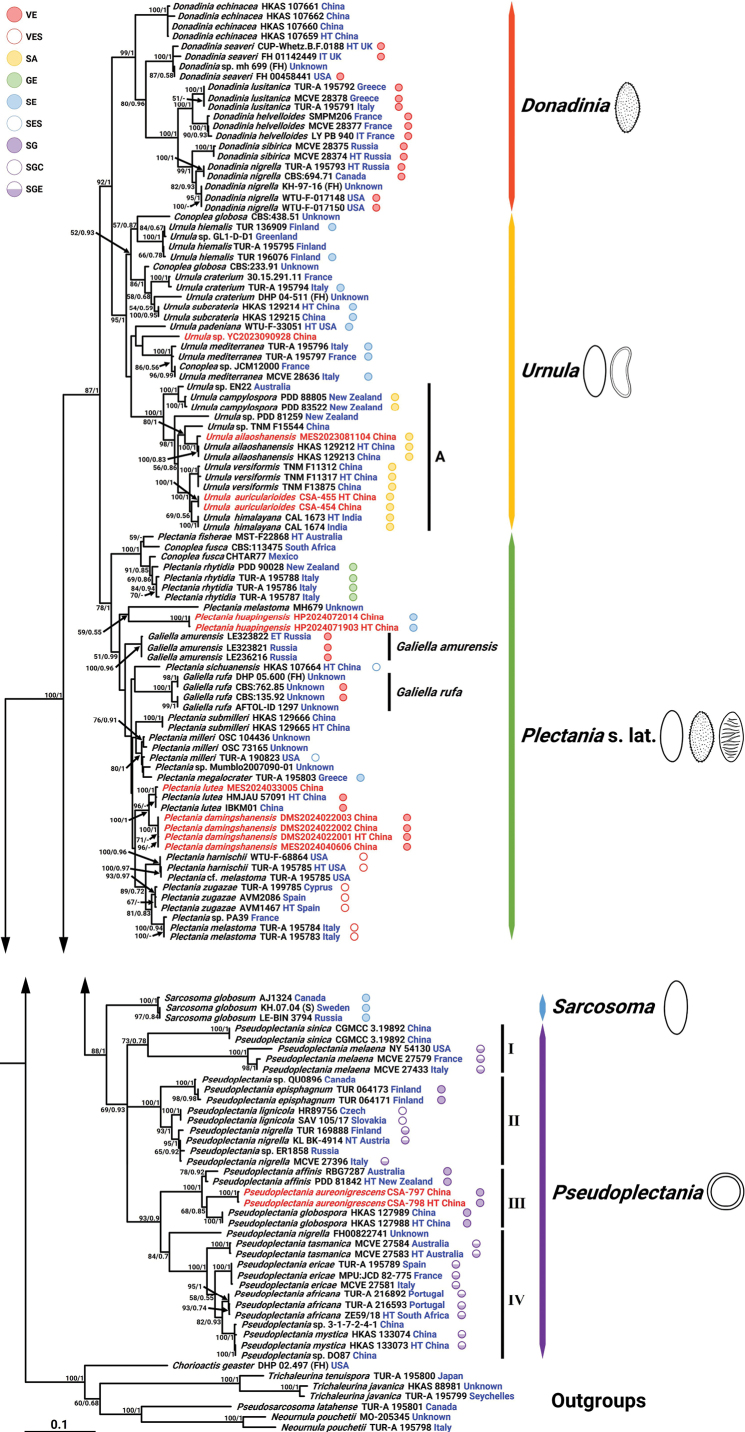
Phylogenetic tree of Sarcosomataceae inferred from the dataset with the aligned ITS + nrLSU + *rpb1* + *rpb2* + *tef1-α* contig. Nodes are separated by ‘-/-’ (MLB/BPP) and indicated if bootstrap support values for ML ≥ 50% and/or posterior probability for BI ≥ 0.90. Specimens of new taxa of the current study are highlighted in red. The letters HT, ET, IT, and NT, and the country highlighted in blue after the specimens stand for holotype, epitype, isotype, neotype, and the original country, respectively. Icons depicting the shape and ornamentation of spores and the presence and position of a gelatinous sheath are indicated after the specimens and correspond to abbreviations. VE indicates verrucose or vermicular ellipsoid spores without a gelatinous sheath. VES indicates verrucose or vermicular ellipsoid spores with a gelatinous sheath. SA indicates a smooth allantoid spore without a gelatinous sheath. GE indicates grooved ellipsoid spores without a gelatinous sheath. SE indicates smooth ellipsoid spores without a gelatinous sheath. SES indicates smooth ellipsoid spores with a gelatinous sheath. SG indicates smooth, globose to subglobose spores without a gelatinous sheath. SGC indicates smooth globose to subglobose spores with a centrally arranged gelatinous sheath. SGE indicates smooth globose to subglobose spores with an eccentrically arranged gelatinous sheath.

A total of 3,599 aligned nucleotide sites were used for the phylogenetic reconstruction, including positions 1–501 bp for ITS, 502–1382 bp for nrLSU, 1383–2169 bp for *rpb1*, 2170–3055 bp for *rpb2*, and 3056–3599 bp for *tef1-α*. The best-fit substitution models selected for reconstructing the phylograms were GTR+I+G for ITS, nrLSU, and *tef1-α*; GTR+G for *rpb1*; and SYM+I+G for *rpb2*. The output topology of the Bayesian inference (BI) tree was nearly identical to that of the maximum likelihood (ML) analysis, so only the ML tree is shown in Fig. 1.

In this multi-locus phylogenetic analysis, the family Sarcosomataceae formed a monophyletic group with strong support (MLB = 100%, BPP = 1). The tree also demonstrated that *Donadinia*, *Pseudoplectania*, *Sarcosoma*, and *Urnula* are monophyletic, while *Galiella* and *Plectania* are polyphyletic. Based on the overall topology, five single, well-supported clades were clearly inferred within Sarcosomataceae. These five clades, comprising 137 taxa representing 45 species, were as follows: the *Donadinia* clade (MLB = 99%, BPP = 1), *Plectania* s. lat. clade (MLB = 78%, BPP = 1), *Pseudoplectania* clade (MLB = 69%, BPP = 0.93), *Sarcosoma* clade (MLB = 100%, BPP = 1), and *Urnula* clade (MLB = 95%, BPP = 1). Notably, the *Pseudoplectania* clade was significantly supported as a sister group to the *Sarcosoma* clade (MLB = 88%, BPP = 1). The *Urnula* and *Donadinia* clades formed a monophyletic group (MLB = 92%, BPP = 1), which was sister to the *Plectania* s. lat. clade (MLB = 87%, BPP = 1).

Within the *Plectania* s. lat. clade, the two *Galiella* lineages—*Galiella amurensis* and *G. rufa*—clustered with *Plectania* species. *Plectania damingshanensis* formed a strongly supported lineage (MLB = 100%, BPP = 1) with four specimens and was recovered as sister to *P. lutea*. Meanwhile, HP2024071903 and HP2024072014 formed a relatively independent branch with strong support (MLB = 100%, BPP = 1) and are regarded here as *P. huapingensis*.

In *Pseudoplectania*, based on distinguishing morphological characters (see details in “Discussion”), high statistical support, and clear genetic relationships, a further division is proposed here: subclade I (MLB = 79%, BPP = 0.78), subclade II (MLB = 100%, BPP = 1), subclade III (MLB = 100%, BPP = 1), and subclade IV (MLB = 84%, BPP = 0.7). A lineage labeled *Pseudoplectania aureonigrescens* was strongly supported (MLB = 100%, BPP = 1) with two specimens and was affiliated with *Ps. affinis* and *Ps. globospora* within subclade III.

In *Urnula*, subclade A was significantly supported (MLB = 100%, BPP = 1). Within subclade A, the lineage representing *U. auricularioides* (MLB = 100%, BPP = 1) grouped together with *U. himalayana*. The topology also showed that MES2024033005 belongs to *P. lutea*, while MES2023081104 belongs to *U. ailaoshanensis*, and YC2023090928 was recovered in the genus *Urnula*.

### ﻿Taxonomy

#### ﻿ Plectania damingshanensis

Taxon classificationFungiPezizalesSarcosomataceae

G. F. Mou & J. R. Liu sp. nov.

4161D2E7-42F1-5803-A5E5-D31224133D0D

Fungal Names: FN 572018

Figs 2 , 3 , 4

##### Etymology.

The specific epithet refers to the type locality, Damingshan National Nature Reserve, in China.

##### Diagnosis.

It is characterized by the cupulate ascomata, the surface of hymenium with brownish orange to light brown color, external surface and stipe (when present) densely covered with grey tomentum and tree-like ridges, the elliptical ascospore is warty under SEM, (21.7) 22.5–32.0 (34.6) × (11.0) 12.1–14.7 (16.5) μm.

##### Holotype.

China • Guangxi Zhuang Autonomous Region, Nanning City, Damingshan National Nature Reserve, 23°49'N, 108°43'E, ca 1230 m alt., on rotten fallen sticks or wood surrounded by moss in the evergreen broad-leaved forest, 19 Feb. 2024, Guang R. Zhou (DMS2024022001, IBK, holotype!) (ITS: PP864720; nrLSU: PP859016; *rpb*1: PV247118).

##### Description.

**Figure 2.**
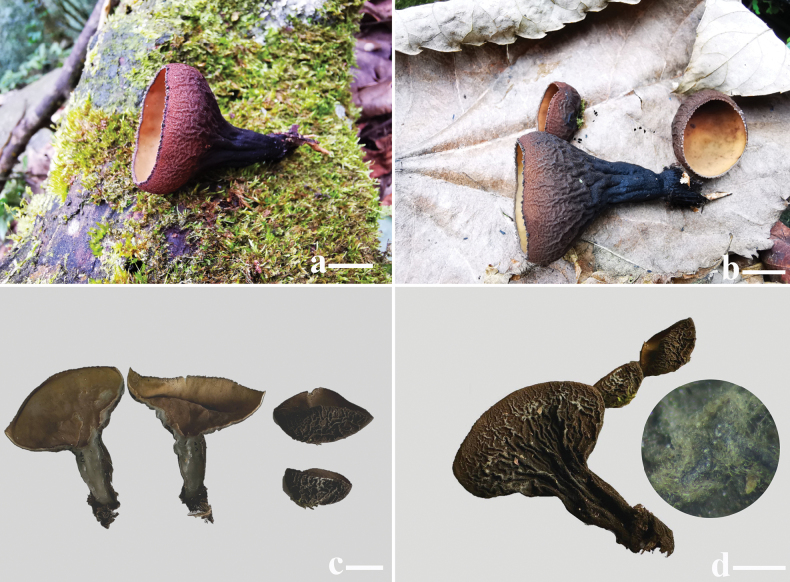
Ascocarps of *Plectania damingshanensis*. Photos by Guang R. Zhou and G. F. Mou. Scale bars: 10 mm (a–d).

***Apothecium*** cupulate, up to 19–27 mm in diameter, 20–30 mm high, subsessile to stipitate (Fig. 2a, b). ***Hymenium surface*** glabrous, without ornamentation or wrinkled, brownish orange (6C6) to light brown (6D7), brown-dark, brown (6E8–6F8) after drying, margin with light brown (6D5) tomentum. ***External surface*** reddish brown-dark brown (7D8–7F8), brownish grey (8F2) after drying. ***Stipe* (when present)** up to 2–20 mm long, 7.5–9 mm in width, densely covered with grey (8D1–8F1) tomentum and tree-like ridges extending all the way to the margin of the cup (Fig. 2b, d). ***Flesh*** solid, wet, gelatinous at a high level, semitransparent, slight grey (8B1). ***Odor* and *taste*** not special.

**Figure 3.**
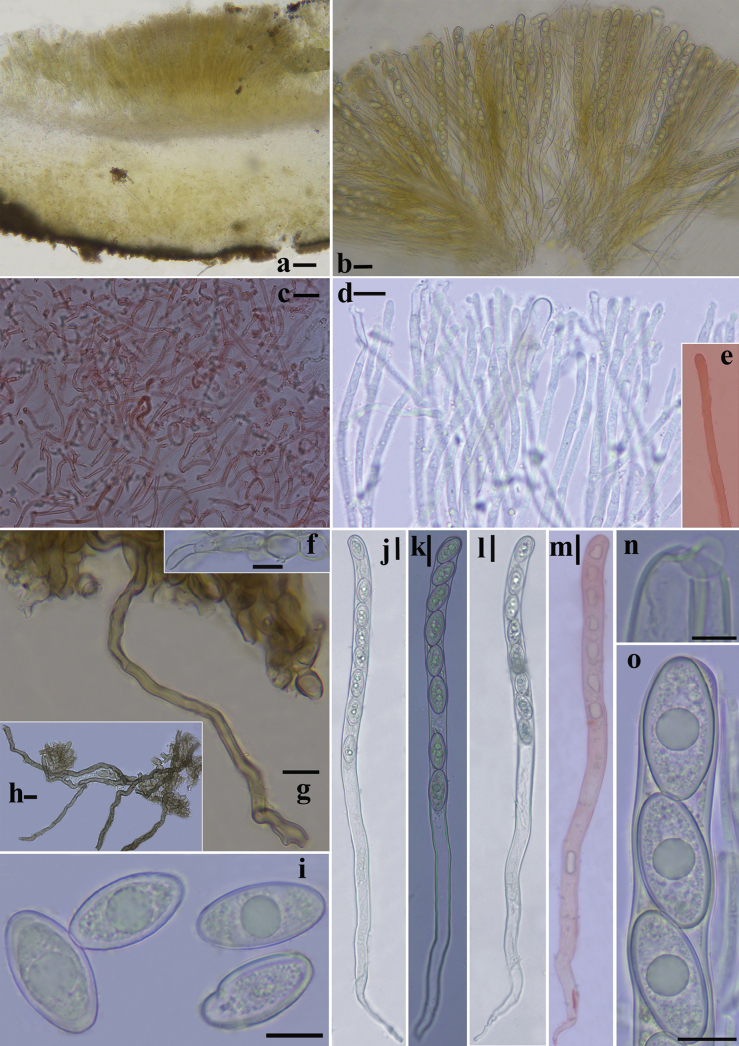
Features of *Plectania damingshanensis*. (photos by J. R. Liu, from holotype: DMS2024022001, IBK!). a. Section of apothecium; b. Hymenium; c. Medullary excipulum; d. Paraphyses and a hymenial hair; e. Hymenial hair; f. Moniliform hair-like cells; g, h. External hairs; i. Ascospores; j–m, o. Asci; n. Apex of asci. Scale bars: 100 μm (a); 20 μm (b, j–m); 10 μm (c–i, n–o). c, e, m in 1% Congo Red solution.

**Figure 4.**
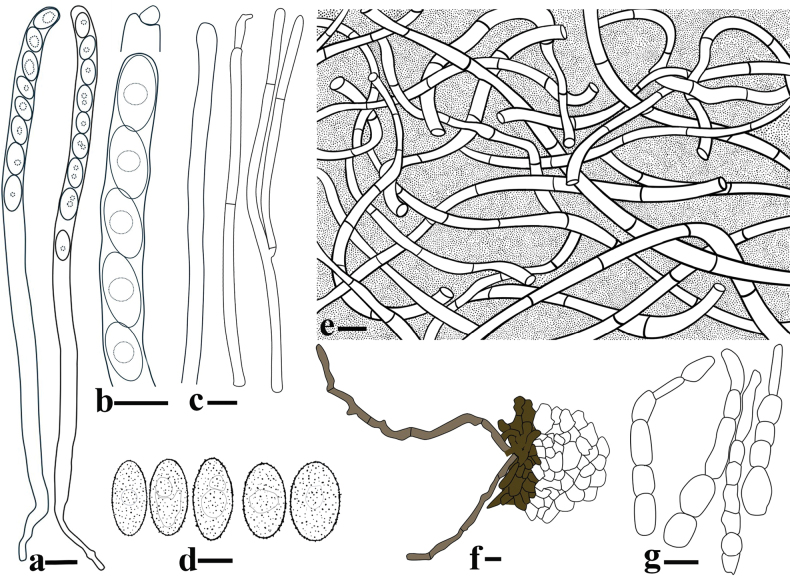
Microscopic structures of *Plectania damingshanensis* (drawings by J. R. Liu, from holotype DMS2024022001, IBK!). a. b. Asci; c. Hymenial hair; d. Paraphyses; e. Ascospores; f. Medullary excipulum; g. External hairs and ectal excipulum; h. Moniliform hair-like cells. Scale bars: 20 μm (a, b); 10 μm (c, d, f–h); 5 μm (e).

***External hairs*** curved and flexuous, septate, glabrous, brown (6E7) with an olive tint (1E8), 5–8.5 μm in diameter, with obtuse end (Fig. 3g, h). ***Ectal excipulum*** of *textura angularis* made up of thick-walled cells, brownish to dark brown, 13–91 μm thick (Fig. 3g), near the margin arranged in separable moniliform hair-like cells, 6.2–10.6 μm diam (Figs 3f, 4g). ***Medullary excipulum*** of loose *textura intricata* immersed in a highly gelatinous matrix, approximately 380–1000 μm, subhyaline to yellowish white (2A2), composed of 2–4.2 μm broad hyphae (Figs 3c, 4f). ***Subhymenium*** of a dense *textura intricata* of closely septate hyphae, olive brown (4D7–4D6), 50–80 μm thick. ***Asci*** cylindrical, suboperculate, 8-spored, rarely with 7 spores, thick-walled, inamyloid, 322–401 (426) × 11.0–15.6 (Fig. 3j–m, 4a). ***Paraphyses*** 1.5–3 μm diam, filiform, septate, branched, slightly enlarged or narrowed near apex (Figs 3d, 4d). ***Ascospores*** uniseriate, ellipsoid, equilateral, hyaline, very finely warty under light microscope (×1000, Fig. 3i), verrucose to vermicular under scanning electron microscope (Fig. 16b), with 1–2 guttules, [40/5/2] (21.7) 22.5–32.0 (34.6) × (11.0) 12.1–14.7 (16.5) μm, Q = (1.7) 1.8–2.3 (2.6), Q_av_ = 2.0. ***Hymenial hairs*** cylindrical, comparable in length to the paraphyses, non-septate, 2.1–4.4 μm in diameter, concolor with paraphyses due to the homogeneous pigments, agglutinating or intermingle with paraphyses to bundles, tips rounded to slightly subcapitate, straight or curved, unbranched (Figs 3e, 4c). ***Anamorph*** unknown.

##### Habitat.

Growing in scattered to gregarious groups on moss-covered decaying wood in broadleaf forests.

##### Geographic distribution.

So far, only known from Guangxi Zhuang Autonomous Region, China.

##### Other specimens examined.

China • Damingshan National Nature Reserve, 23°49'N, 108°43'E, alt. 1225 m, on wood surrounded by moss in the evergreen broad-leaved forest, 19 Feb. 2024, Guang R. Zhou, DMS2024022002 (IBK!) (ITS: PP864721; nrLSU: PP859017; *tef1*-*α*: PV295998); DMS2024022003 (IBK!) (ITS: PP864722; nrLSU: PP859018). Mao’ershan National Nature Reserve, 25°88'N, 110°37'E, ca 720 m alt., on rotten fallen sticks, 6 April 2024, Yan C. Zhang, MES024040606 (IBK!) (ITS: PQ691392; nrLSU: PQ682446; *rpb1*: PV247119; *tef1*-*α*: PV295999).

##### Notes.

In the phylogenetic tree (Fig. 1), *P. damingshanensis* and *P. lutea* were grouped together with strong support (MLB = 100%, BPP = 1). Both species share ellipsoid and verrucose ascospores with similar dimensions: *P. damingshanensis* measures (21.7) 22.5–32.0 (34.6) × (11.0) 12.1–14.7 (16.5) μm, while *P. lutea* measures (24.5) 27.0–30.0 (32.0) × (12.2) 13.0–15.0 (16.0) μm (Mou and Bau 2021). However, the external surface of *P. damingshanensis* is densely covered with grey tomentum and tree-like ridges that extend to the margin, and its apothecium is larger with a longer and thicker stipe. While the external surface of *P. lutea* has fewer brown hairs and irregular, vascular ridge-like protrusions, with ascomata measuring only 11.0–18.0 mm in diameter. *P. sichuanensis* is distinguished from *P. damingshanensis* by its hymenium, which is ochre in color and lacks a noticeable covering of black hairs, and its smaller, smooth ascospores (22–26 × 11–13 μm) (Zeng et al. 2021).

From a microscopic perspective, the ascospores of *P. damingshanensis*, as well as those of *P. zugazae* Calonge & Alb. García, *P. melastoma* described in older mycological literature, and *P. harnischii* M. Carbone, Agnello, A.D. Parker & P. Alvarado, are all elliptical and verrucose (Carbone et al. 2015a, 2021). However, there are significant differences in ascospore size among the four species, *P. damingshanensis* having larger ascospores [(21.7) 22.5–32.0 (34.6) × (11.0) 12.1–14.7 (16.5) μm], while ascospores of *P. zugazae* [18–22 × 12–14 μm] and *P. melastoma* [21.8–25 × 10–12.4 μm] are notably smaller. As for *P. harnischii*, it exhibits the narrower ascospores with a size of 20.2–24.8 × 8.1–11.2 µm.

*Plectania damingshanensis* exhibits numerous similarities to species within the *Galiella* genus, featuring brightly colored ascocarps, highly gelatinized fleshy tissue, and ascospore surfaces adorned with wart-like ornamentation. However, on the phylogenetic tree, *P. damingshanensis* does not cluster with *G. amurensis* and *G. rufa*. Additionally, it is noteworthy that the ascospores of *G. amurensis* lack oil droplets, and its asci (measuring 400–450 × 12–17 μm) and ascospores [measuring (26) 28–37.5 (40) × 11.5–17.5 (18) μm] are larger compared to those of *P. damingshanensis*. Conversely, the asci (measuring 270 × 12 μm) and ascospores [measuring (17) 18–21 (23) × 8–10 μm] of *G. rufa* are smaller (Carbone et al. 2015b).

#### ﻿ Plectania huapingensis

Taxon classificationFungiPezizalesSarcosomataceae

G. F. Mou & J. R. Liu sp. nov.

B0BAC2CA-E50F-5A9E-826D-898D31D8E508

Fungal Names: FN 572466

Figs 5 , 6 , 7

##### Etymology.

The specific epithet refers to the type locality, Huaping National Nature Reserve, in China.

##### Diagnosis.

It is characterized by having ascomata, are clustered, disc-shaped, or margin irregular disc-shaped, and the outer surface of the apothecium is adorned with brown-orange to brownish-red particles; under scanning electron microscope (SEM) observation, the elliptical ascospores have a smooth surface, with a size of 17.3–21.4 (23.1) × 8.1–10.9 (11.7) μm, and 7- or 8-spored asci with a size of 363–388 (425.8) × 9.2–14.1 μm.

##### Holotype.

China • Guangxi Zhuang Autonomous Region, Guilin City, Huaping National Nature Reserve, 25°63'N, 109°92'E, ca 950 m alt., on buried wood on the ground mixed with evergreen broad-leaved trees and bamboo, 19 July 2024, J. R. Liu (HP2024071903, IBK, holotype!) (ITS: PQ691391; nrLSU: PQ682445; *rpb2*: PV296007; *tef1*-*α*: PV296000).

##### Description.

**Figure 5.**
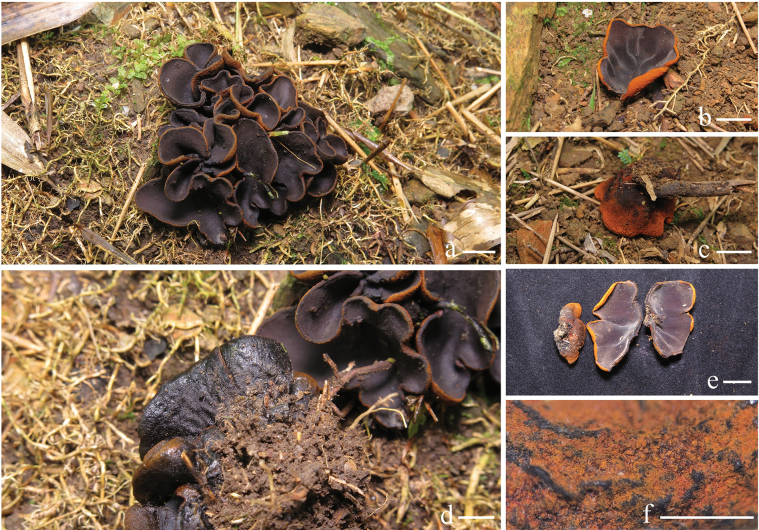
Ascocarps of *Plectania huapingensis* (photos by J. R. Liu). a, d. Habitat of specimen HP2024071903 (IBK!), found on senescing or dead bamboo rhizomes; b, c. Habitat of specimen HP2024072014 (IBK!), growing on dead woody branches on the ground or dead bamboo rhizomes; e. Vertical section showing dark violet thin flesh; f. Details of the external surface. Scale bars: 10 mm (a–e); 1 mm (f).

**Figure 6.**
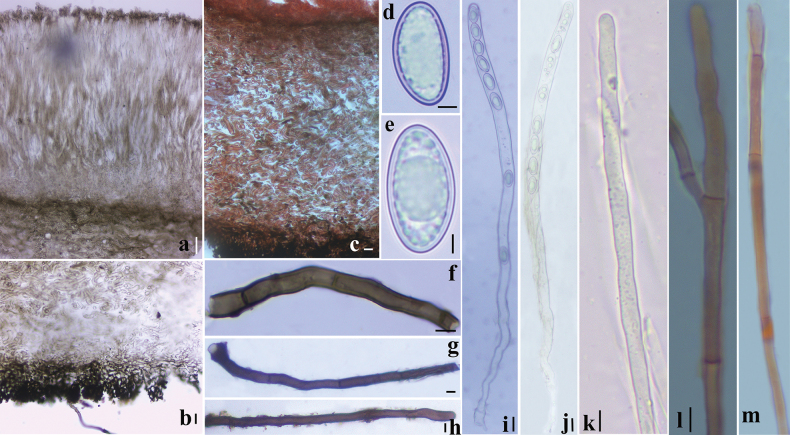
Features of *Plectania huapingensis* (photos by J. R. Liu, from HP2024071903, IBK!). a. Section of apothecium; b. External hairs; c. Medullary excipulum; d, e. Ascospores; f–h. External hairs; i, j. Asci; k. Hymenial hair; l, m. Paraphyses. Scale bars: 50 μm (a); 10 μm (b, c, i–m); 5 μm (d–h).

***Apothecium*** clustered, sometimes single, disc-shaped or margin irregular disc-shaped, reaching a diameter of 18–26 mm, with a thickness of 1 mm, subsessile (Fig. 5a). ***Hymenium surface*** has wart-like protrusions, some of which are adorned with tiny tomentum, dark brown (Fig. 5b, e). ***External surface*** shares a similar color to the hymenium, being relatively rough and adorned with brownish-orange to brownish-red particles, which are typically confined to the cup margin but occasionally cover the entire outer surface, relatively tough in texture (Fig. 6c, d). ***Odor* and *taste*** not special.

**Figure 7.**
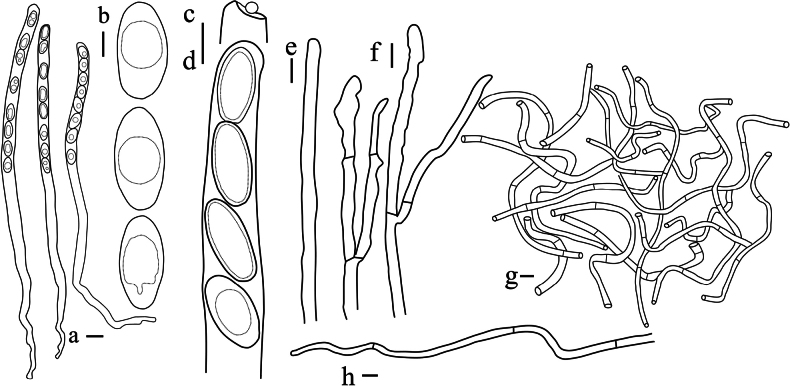
Microscopic structures of *Plectania huapingensis* (drawings by J. R. Liu). a, d. Asci; b. Ascospores; c. Apex of asci; e. Hymenial hair; f. Paraphyses; g. Medullary excipulum; h. External hairs. Scale bars: 10 μm (a, c, d); 5 μm (b, e–h).

***External hairs*** are nearly cylindrical, septate, with a diameter ranging from (5.1) 6.0–7.2 (7.5) μm, slightly curved, and smooth. They originate from the outer cortical layer and appear brown due to epidermal pigmentation, with walls thickened up to 1 μm (Figs 6f–h, 7h). ***Ectal excipulum*** consists of a *textura angularis*, thick-walled cells that are dark brown and measuring 115–135 μm, with slightly thick walls. ***Medullary excipulum*** composed of a *textura intricata*, measuring 480–550 μm in thickness, subhyaline to yellowish white, and consisting of hyphae 4.1–7.0 μm in diameter (Figs 6c, 7g). ***Subhymenium*** is a dense *textura intricata* of closely septate hyphae, olive-colored (2F5), with a thickness of (46.6) 62.2–110 μm. ***Hymenium*** (329.5) 363.5–422.8 μm thick. ***Asci*** cylindrical, suboperculate, 8-spored (occasionally with 7 spores), thick-walled, inamyloid, measuring 363–388 (425.8) × 9.2–14.1 μm (Figs 6i, j, 7a). ***Paraphyses*** 1.9–3.4 μm in diameter, filiform, septate, branched, and slightly enlarged or narrowed near the apex (Figs 6l–m, 7f). ***Ascospores*** uniseriate, ellipsoid, equilateral, hyaline, smooth, usually contain 1–2 guttules and measuring [37/6/2] 17.3–21.4 (23.1) × 8.1–10.9 (11.7) μm (Figs 6d, e, 7b), with Q _values_ = (1.5) 1.7–2.2 (2.4) and an average Q _value_ of 2. ***Crystals*** few to abundant, present among the hairs and in the outer surface of the ectal excipulum. ***Hymenial hairs*** cylindrical, comparable in length with paraphyses, non-septate but with a single septum in the basal part, 2.2–3.3 μm wide, concolor with paraphyses due to the homogeneous pigments, tips rounded, straight to slightly curved, unbranched (Figs 6k, 7e). ***Anamorph*** unknown.

##### Habitat.

Half-buried in the litter layer in moist forest mixed with evergreen broad-leaved trees and bamboo.

##### Geographic distribution.

By now, only known from Guangxi Zhuang Autonomous Region, China.

##### Other material examined.

China • Guangxi Zhuang Autonomous Region, Guilin City, Huaping National Nature Reserve, 25°63'N, 119°92'E, ca 850 m alt., 20 July 2024, J. R. Liu, HP2024072014 (ITS: PQ691390, nrLSU: PQ682444).

##### Notes.

*P. huapingensis* and *P. melastoma* share macroscopic characteristics of apothecial external surfaces and margins covered with orange granules. However, upon comparing their microscopic features, it becomes evident that the asci of *P. huapingensis* [asci: 363–388 (425.8) × 9.2–14.1 μm] and its ascospores [17.3–21.4 (23.1) × 8.1–10.9 (11.7) μm] are smaller than those of *P. melastoma* (asci: 400–450 × 12–15 μm; ascospores: 21.8–25 × 10–12.4 μm) (Agnello and Carbone 2012; Zeng et al. 2021). This difference serves as a distinguishing factor between the two species. Furthermore, with the distinctive feature of its apothecial external surface and margin being covered with orange granules, *P. huapingensis* can be clearly differentiated from other species within the genus *Plectania*.

*P. huapingensis* shares morphological similarities with *Korfiella karnika*D.C. Pant & V.P. Tewari in both macroscopic and microscopic characteristics. However, molecular sequence data from *K. karnika* and its allied species remain unavailable. Given that *P. huapingensis* aligns with the genus *Plectania* in molecular phylogeny and micromorphological features, *P. huapingensis* exhibits morphological distinctions from *K. karnika*, specifically the presence of hymenial hairs (absent in *K. karnika*) and mature asci containing 7–8 ascospores (versus 2–4 ascospores per ascus in *K. karnika*), and it should be classified as a member of *Plectania* s. lat clearly from phylogenetic evidence (Pant and Tewari 1970).

#### ﻿ Plectania lutea

Taxon classificationFungiPezizalesSarcosomataceae

T. Bau & G. F. Mou, Phytotaxa, 509(3), 288. 2021.

2CB95315-BA7F-52B8-80BB-120400965FD1

Fig. 8

##### Material examined.

China • Guangxi, Guilin city, Mao’ershan National Nature Reserve, 25°87'N, 110°47'E, ca 540 m alt., on rotten fallen sticks, 30 Mar 2024, J. R. Liu (MES2024033005, IBK!) (ITS: PP859394; nrLSU: PP981371; *rpb1*: PV247120; *rpb2*: PV296006; *tef1-α*: PV296001).

##### Geographic distribution.

Currently known in Guangxi Zhuang Autonomous Region in China.

**Figure 8.**
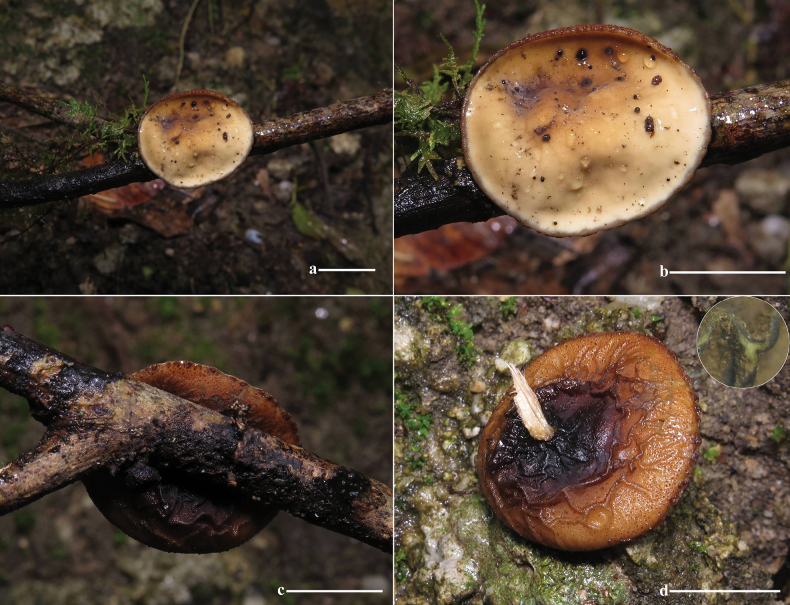
Ascocarps of *Plectania lutea*, collected from the Mao’ershan National Nature Reserve (photos by J. R. Liu). a–c. Habitat of specimen MES2024033005 (IBK!), growing on dead woody branches on the ground; d. External surface with a detail of the short hirsute hairs. Scale bars: 10 mm (a–d).

##### Notes.

The morphological characteristics of this specimen are identical to the original description of *P. lutea* provided by Mou and Bau (2021).

### ﻿Taxonomic Keys for Species Classification of *Plectania* in China

**Table:** 

1	Ascospores ellipsoid	**2**
–	Ascospores inequilateral ellipsoid with a flattened side	**3**
2	Ascospores surface smooth	**4**
–	Ascospores surface covered with verrucose ornamentation	**5**
3	Ascospores one side bearing 13 to 17 transverse grooves, 19.6–27.4 (29.4) × (6.9) 7.8–12.3 μm	***P. rhytidia***
–	Ascospores one side bearing 8 to 12 rib-like horizontal furrows, 18–22 × 10–12 μm	***P. platensis***
4	Apothecium sessile, ascospores relatively large, 35–42 × 13–18 μm	***P. yunnanensis***
–	Apothecium substipitate, ascospores smaller	**6**
5	Apothecium11.0–18.0 mm, with short stipe, external surface sparsely covered with tomentum	***P. lutea***
–	Apothecium19–27 mm, with longer and thicker stipe, external surface densely covered with grey tomentum	***P. damingshanensis***
6	Apothecia external surface and margin covered by orange granules	**7**
–	Apothecia external surface covered or not by orange or brownish granules often restricted at the margin	***P. sichuanensis***
7	Asci and ascospores relatively large, asci measuring 400–450 × 12–15 μm, ascospores measuring 21.8–25 × 10–12.4 μm	***P. melastoma***
–	Asci and ascospores relatively small, asci measuring 363–388 (425.8) × 9.2–14.1 μm, ascospores measuring 17.3–21.4 (23.1) × 8.1–10.9 (11.7) μm	***P. huapingensis***

#### ﻿ Pseudoplectania aureonigrescens

Taxon classificationFungiPezizalesSarcosomataceae

S. A. Chen, D. Li & G. F. Mou sp. nov.

A97FD638-D978-56C4-AB24-BE34E16F75D4

Fungal Names: FN 572467

Figs 9 , 10 , 11

##### Etymology.

The specific epithet “*aureonigrescens*” refers to the yellowish to orangish color of hymenium surface when young, which changes to grayish black at maturity.

##### Diagnosis.

Differs from other known *Pseudoplectania* species by combination of the following features: sessile ascomata, bowl-shaped, petal-shaped to irregular apothecium up to 25 mm in diameter, hymenium surface yellow-colored when young, changing to grey or black when mature; one type of external hairs straight to distinctly curved or flexuous (not coiled), paraphyses with a mostly straight and rarely diverticulated tip, crystals in the hymenium up to 19.5 μm wide, and smooth ascospores without a gelatinous sheath.

##### Holotype.

China • Fujian Province, Fuzhou City, Cangshan District, Fujian Agriculture and Forestry University, Baizhu Garden, 26°08'N, 119°24'E, ca 50 m alt., on senescing to dead rhizomes of bamboo (probably *Oligostachyum lubricum*) exposed outside mossy soil walls in a planted bamboo forest, 29 Oct. 2024, D. Li & S. A. Chen (CSA-798, IBK, holotype!) (ITS: PQ895821; nrLSU: PQ863683; *rpb1*: PV247121; *tef1-α*: PV296005).

##### Description.

**Figure 9.**
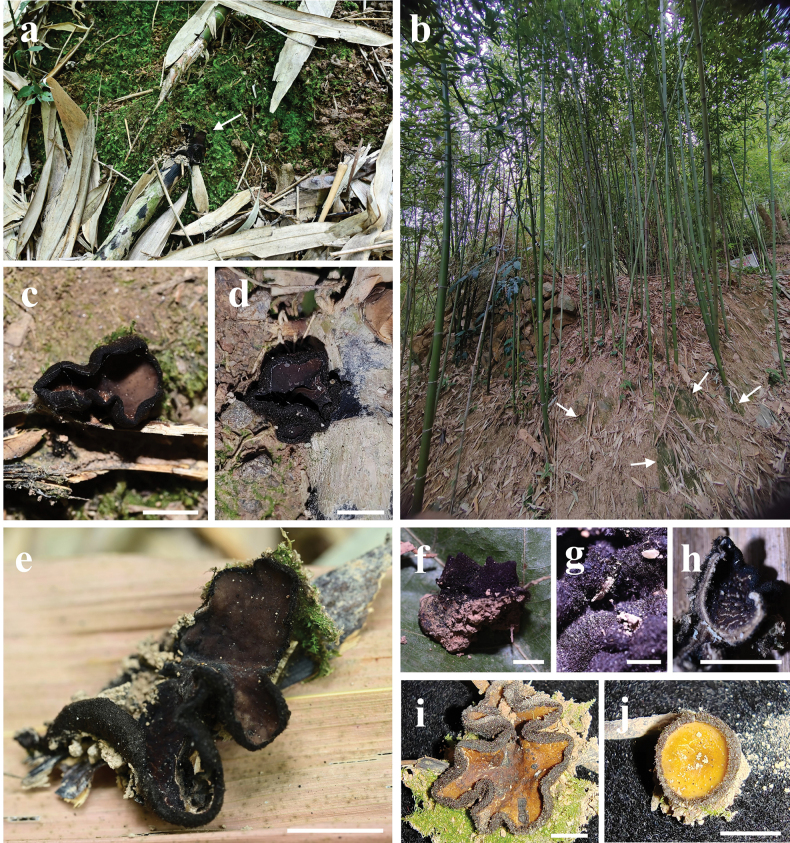
Ecological and macroscopic characteristics of *Pseudoplectania aureonigrescens*, photos by S. A. Chen & D. Li; c–e. from holotype CSA-798 (IBK!); f–j. from CSA-797 (IBK!). a. Habitat where the specimen CSA-798 (IBK!) grew (the white arrow indicates the growth site of ascomata), senescing or dead rhizomes of bamboo exposed outside mossy soil walls; b. Habitat where the specimen CSA-797 (IBK!) grew (white arrows indicate growth sites of ascomata), a planted bamboo forest; c–e. Mature ascomata with a smooth, greyish-black hymenium; f. Off-black external surface covered with abundant tomenta; g. Details of the hairy external surface; h. Vertical section including greyish thin flesh; i, j. Young ascomata with discoid to petal apothecium, dirty orange hymenium, and velvety to strigose external surface. Scale bars: 10 mm (c, e); 5 mm (d, f, h–j); 2 mm (g).

***Ascomata*** tiny to small, sessile. ***Apothecium*** bowl-shaped, petal-shaped to irregular, 5–24 mm in diameter, up to 10 mm high; margin entire and involute, cracked after drying. ***Hymenium surface*** yellow-tan (2A6), dirty orange (4B8) to golden brown (5C7) when young, glabrous, dull, plum grey (8E5), wine grey (9E3) to off-black (4F8) at maturity, color unchanging when bruised, cracked after drying. ***External surface*** velvety to strigose, off-black (4F8), densely covered with tomentum or short bristles, without ridges or crests. ***Stipe*** absent (Fig. 9f). ***Subiculum*** off-black (4F8), cotton-like, dense, soft. ***Flesh*** thin, conch grey (22B1), cloud grey (15C1) to pewter grey (10E1). ***Odor* and *taste*** not special. ***Anamorph*** not observed.

**Figure 10.**
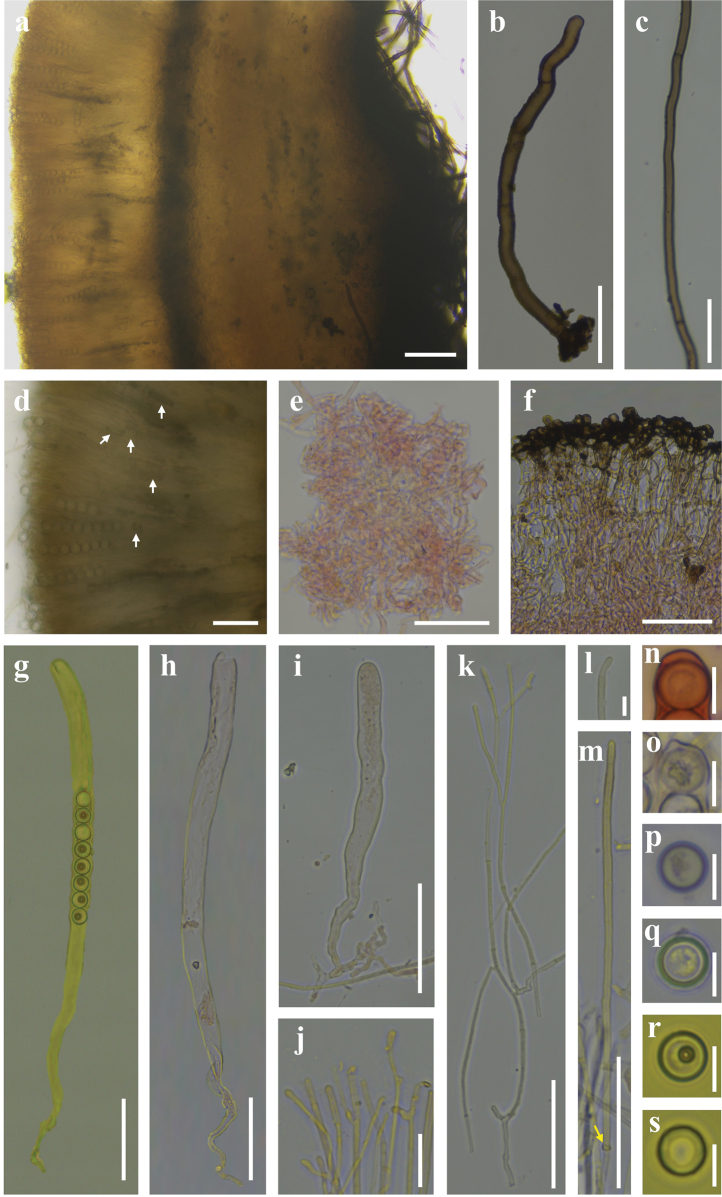
Microscopic structures of *Pseudoplectania aureonigrescens*, photos by S. A. Chen, from holotype CSA-798 (IBK!). a. Cross section through apothecia; b. External hair; c. Hypha of subiculum; d. Hymenium with crystals (white arrows indicate the position of crystals); e. Medullary excipulum in Congo red; f. Ectal excipulum in Congo red; g. Ascus with mature ascospores in Meltzer’s reagent; h. Empty ascus with opened operculum in Congo red; i. Immature ascus without visible ascospores in Congo red; j, k. Paraphyses in Congo red; l, m. Hymenial hair in Congo red (the yellow arrow in m indicates the basal septa of the hymenium hair); n. Rounded apex of asci in Congo red; o. Apex of asci with a conical eccentric protrusion; p, q. Ascospores in distilled water; r, s. Ascospores in Meltzer’s reagent. Scale bars: 100 μm (a); 50 μm (b–i, k, m); 20 μm (j); 10 μm (l, n–s).

**Figure 11.**
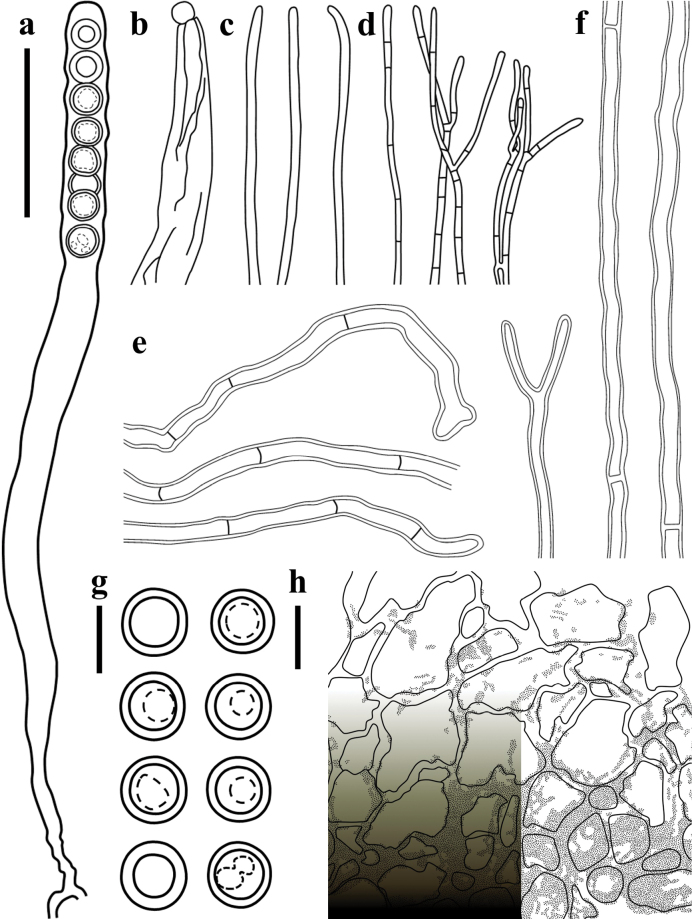
Microscopic structures of *Pseudoplectania aureonigrescens*, drawing by S. A. Chen, from holotype CSA-798 (IBK!). a. An ascus with 8 ascospores; b. Empty ascus with its operculum; c. Hymenium hairs; d. Paraphyses; e. External hairs; f. Hypha of subiculum; g. Ascospores; h. A part of ectal excipulum. Scale bars: 50 μm (a–f sharing same bars); 10 μm (g, h).

***External hairs*** of one type, hyphoid, septate, cylindrical, straight to distinctly curved or flexuous (not coiled), brass brown (1C7), 4–9.5 μm in diameter, with slightly thick to thick walls, up to 2 μm thick, tips rounded, sometimes split to two protuberances, unbranched, surface smooth to slightly rough (Figs 10b, 11e). ***Ectal excipulum*** of a *texture angularis*, made up of properly to distinctly thick-walled (up to 3 μm) cells, measuring 5.5–23 × 3.5–13 μm, sub-hyaline, tawny (4C5), masala chai brown (4D7) to dark brown (5E8), and dull black (18F8), not or very slightly encrusted, thick-walled, up to 180 μm thick (Figs 10a, f, 11h). ***Medullary excipulum*** of loose *textura intricata* immersed in a gelatinous matrix, up to 335 μm thick, with hyphae septate, subhyaline or pale yellow (1A5), thin-walled, flexuous, branched, 2–4 (6) μm wide, tawny (4C5) to light brown (4C7), dark coffee brown (4E8) at low magnification (×100, Fig. 10a, e). ***Subhymenium*** of a dense *textura intricata* of septate hyphae, 2–4 μm wide, thin-walled, concolor with medullary excipulum, up to 80 μm thick, dark coffee brown (4E8) to dark brown (5E8) at low magnification (Fig. 10a). ***Asci*** cylindrical, operculate, with a curved or flexuous, tapered base, 8-spored, 195–335 × 10.5–14 μm, constricts at the junction of two ascospores, non-amyloid, apex mostly rounded, sometimes with a conical, eccentric, or non-eccentric protrusion (Figs 10g–i, n, o, 11a, b). ***Ascospores*** uniseriate, smooth, with thick walls, globose to subglobose, sub-hyaline to pale yellow (1A5), [60/4/2] (8.5) 9.5–11.5 (14) × (8.5) 9–11 (14) μm, Q _value_ = (0.95) 1–1.1 (1.13) including the spore wall, non-amyloid, with or without one to multiple, globose to subglobose contents (Figs 10p–s, 11g). ***Paraphyses*** abundant, filiform, not or slightly extending the length of asci, septate, subcylindrical, simple to bifurcate, straight to slightly curved, sometimes anastomosed, branched both from the lower part and tips, dark khaki brown (5D6) to dark coffee brown (4E8) due to amorphous pigments, (1) 1.5–3 μm in diameter, tips rounded to finger-shaped, rarely with few protuberances or notches, mostly straight, sometimes slightly enlarged or curved, unbranched or branched 1–2 times (Figs 10j, k, 11d). ***Hymenial hairs*** cylindrical, long as the paraphyses, non-septate but with a single septum in the basal part, (1.5) 2–3.5 μm in diameter, concolor with the paraphyses due to the homogeneous pigments, tips rounded, straight to slightly curved, unbranched (Figs 10l, m, 11c). ***Subiculum*** dark coffee brown (4E8) to dark brown (5E8), septate, thick-walled, straight to curved or flexuous, mostly unbranched, rarely branched once, not encrusted, 4–6.5 μm in diameter, with a rounded tip (Figs 10c, 11f). ***Crystals* in the *hymenium*** rectangle to angular, sometimes cracked, roughly parallel to other elements in hymenium, concolor with the paraphyses, mainly of two types, although intermediates forms may exist: 1) thin, 1.9–4.5 μm in diameter, up to 24.5 μm long; 2) thick, 7–19.5 μm in diameter, up to 27.5 μm long (Fig. 10d).

##### Habitat.

Growing in scattered to gregarious groups on senescing to dead rhizomes of bamboo (probably *Oligostachyum lubricum*) exposed outside mossy soil walls in bamboo forests.

##### Geographic distribution.

So far, only known from Fujian Province, China.

##### Other material examined.

China • Fujian Province, Fuzhou City, Cangshan District, Fujian Agriculture and Forestry University, Baizhu Garden, 26°08'N, 119°24'E, ca 50 m alt., on senescing to dead rhizomes of bamboo exposed outside mossy soil walls in a planted bamboo forest (probably *Oligostachyum lubricum*), 12 Nov. 2023, S. A. Chen, CSA-797 (IBK!) (ITS: PQ863322; LSU: PQ863491; *tef1-α*: PV296004).

##### Notes.

In the phylogenetic analyses, *Pseudoplectania aureonigrescens* clustered with *Ps. affinis* and *Ps. globospora* in clade III. The sessile ascomata with entire margin, velvety, rough, blackish external surface, dark hymenium surface at maturity, and globose to subglobose ascospores without gelatinous sheath make them easily confused (Carbone et al. 2014a; Zeng et al. 2024). But it can still be recognized by some distinguishing characters. With respect to *Ps. affinis*, *Ps. aureonigrescens* has variously shaped apothecium, dull hymenium surface at maturity, hymenium with crystals, existing obviously curved or flexuous external hairs, and paraphyses rarely with protuberances or notches. Compared with *Ps. globospora*, *Ps. aureonigrescens* can be distinguished by its variously shaped apothecium, one-typed external hairs, smaller cells in ectal excipulum (5.5–23 × 3.5–13 μm *vs.* 17−22 × 14−17 μm), existing crystals in hymenium, slightly smaller ascospores (9.5–11.5 × 9–11 μm *vs.* 10.5–12.5 × 9.8–13.5 μm), and also by its lignicolous habitat. Also, the lineage belongs to the newly established species, obtains strong support, and is enough to separate itself from its relatives by genetic distance.

Ecologically, *Pseudoplectania aureonigrescens* and *Ps. mystica*, teleomorphs of both, share a similar habitat and are geographically close in distribution, growing groups on senescing to dead rhizomes of bamboo (Lin et al. 2024). It is also notable that *Ps. aureonigrescens* shares similarities with *Ps. mystica* in its sessile ascomata, velvety, dark external surface, crystals in hymenium, existing wavy or flexuous external hairs, paraphyses rarely with protuberances or notches. However, besides its distant of genetic, this new species is clearly differentiated by ascomata of various shapes, dull hymenium surface when mature, existing branched subiculum, a lack of crystals in ectal excipulum, and slightly smaller ascospores without sheath (9.5–11.5 × 9–11 μm *vs.* 11–13 × 10.5–12 μm).

### ﻿Key to accepted species of *Pseudoplectania*

*Pseudoplectania* species lacking molecular data or clear original diagnoses are not included (viz., *Pseudoplectania carranzae*, *P.. kumaonensis*, *P.. spongiosa*, and *P.. stygia*). *P.. sinica* is also excluded due to the lack of teleomorph.

**Table:** 

1	Mature ascospores with slight ornamentation	***Pseudoplectania ryvardenii***
–	Mature ascospores without ornamentation	**2**
2	Mature ascospores with a gelatinous sheath	**3**
–	Mature ascospores without a gelatinous sheath	**9**
3	Mature ascospores with centrally arranged gelatinous sheath; ectal excipulum composed of subglobose to elongated cells	***Ps. lignicola***
–	Mature ascospores with eccentrically arranged gelatinous sheath; ectal excipulum without elongated cells	**4**
4	Ascomata distinctly stipitate; paraphyses tips often hooked	***Ps. melaena* complex**
–	Ascomata sessile to shortly stipitate; paraphyses tips straight to curved, but not hooked	**5**
5	External hairs flexuous or coiled	**6**
–	External hairs straight to curved or flexuous but not coiled	**7**
6	Large crystals absent	***Ps. nigrella* complex**
–	Large crystals present; known from South Africa and Madira island	***Ps. africana***
7	Paraphyses tips mostly bifurcate to trifurcate	**8**
–	Paraphyses tips simple to bifurcate	***Ps. mystica***
8	Apothecium up to 10 mm; crystals absent in hymenium; known from Europe	***Ps. ericae***
–	Apothecium up to 30 mm; crystals present in hymenium; known from Oceania	***P.. tasmanica***
9	Apothecium mostly up to 10 mm; external hairs flexuous or coiled	***P. episphagnum***
–	Apothecium available more than 10 mm; external hairs straight to curved or flexuous, but not coiled	**10**
10	External hairs two-typed	***Ps. globospora***
–	External hairs one-typed	**11**
11	Hymenium surface shiny or polished when mature; external hairs straight to slightly curved; crystal absent in the hymenium	***Ps. affinis***
–	Hymenium surface dull when mature; external hairs straight to distinctly curved or flexuous; crystals present in the hymenium	***Ps. aureonigrescens***

#### ﻿ Urnula ailaoshanensis

Taxon classificationFungiSuctoridaUrnulidae

J. R. Lu, Y. Zhang & Q. Zhao, Phytotaxa 619 (1): 91. 2023.

3DFF131A-0C18-5445-B27A-E04CA09AB366

Fig. 12

##### Material examined.

China • Guangxi Zhuang Autonomous Region, Guilin City, Mao’ershan National Nature Reserve, on rotten fallen sticks surrounded by mosses, ca 1990 m alt., 11 Aug 2023, J. R. Liu (MES2023081104, IBK!) (ITS: PQ187434; nrLSU: PQ187435; *rpb*1: PV247122).

##### Habitat.

On the fallen, rotten wood surrounded by mosses.

##### Geographic distribution.

Currently known in Yunnan Province and Guangxi Zhuang Autonomous Region in China.

##### Notes.

**Figure 12.**
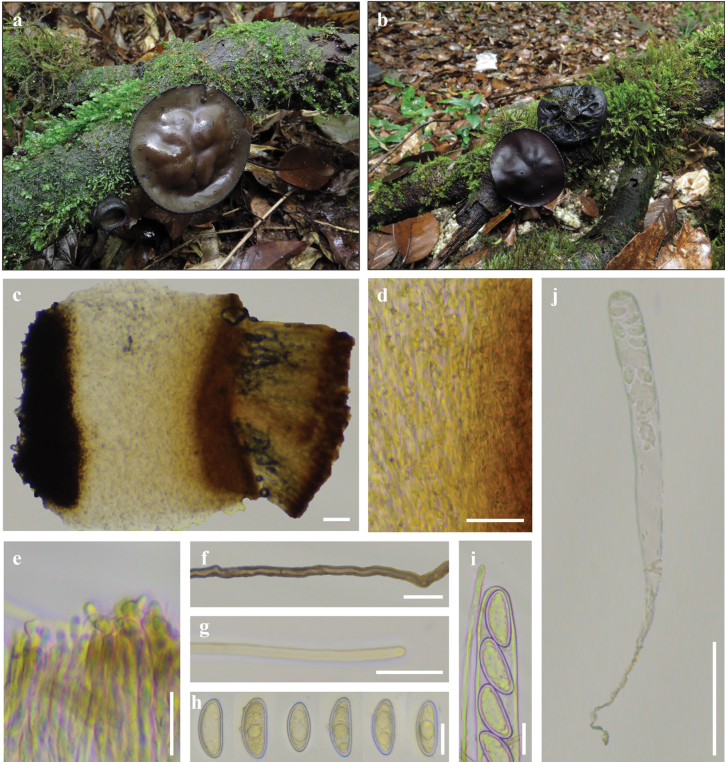
Ecology and macroscopic characteristics of *Urnula ailaoshanensis*, Field photos by J. R. Liu; microscopic photos by S. A. Chen, from IBKMES2023081104 (IBK!). a. Fresh ascomata in a field, growing on rotten wood surrounded by mosses; b. Fresh ascomata with a dark hymenium surface and a reticulate or corrugated external surface; c. Cross Section through apothecia; d. Subhymenium and medullary excipulum; e. Paraphyses; f. External hairs; g. Hymenial hairs; h. Ascospores in water; i. apical apparatus of ascus with developed ascospores; j. An ascus with immature ascospores. Scale bars: 100 μm (c, j); 50 μm (d); 20 μm (f–i); 10 μm (e).

According to Lu et al. (2023), *Urnula ailaoshanensis* was originally reported in Yunnan Province, China, and it is recognized by saucer- or bowl-shaped cup apothecia, reddish-brown to ocher hymenium with glabrous surface, one type of external hair, cylindrical asci with a tapered or attenuated base, and ellipsoid, sometimes reniform-like ascospores. In this study, these features are mostly consistent with the observation of our specimens. However, compared to the type specimens, our specimens have darker hymenium surface, smaller asci (340–450 × 21–26 μm *vs.* 465–600 × 21–30 μm), and slightly longer ascospores (29–38.5 × 13.5–17 μm *vs.* 29–34.5 × 11.5–17 μm). Such variable color of hymenium surface may be associated with the maturity of ascomata and/or environmental factors, which makes *U. ailaoshanensis* easily confused with other *Urnula* species, viz., *U. versiformis* Y. Z. Wang & Cheng L. Huang, *U. himalayana* K. Das & D. Chakr. But *U. ailaoshanensis* can be identified through these microscopic features: one type of external hair, ellipsoid to reniform-like ascospores, and molecular evidence (Wang and Huang 2015; Wang et al. 2018). In addition, we found a few hymenial hairs in the hymenium, cylindrical, non-septate, up to 3.5 μm in diameter, with rounded tips (Fig. 12g).

#### ﻿ Urnula auricularioides

Taxon classificationFungiSuctoridaUrnulidae

S. A. Chen, D. Li & G. F. Mou sp. nov.

C7A1C643-863C-52E9-917F-04E87840E669

Fungal Names: FN 572194

Figs 13 , 14 , 15

##### Etymology.

The specific epithet “*auricularioides*” refers to the appearance and habitat of this species similar to *Auricularia* species.

##### Diagnosis.

Differs from other known *Urnula* species by the combination of the following features: external surface velvety to warty and with ridges, hymenium surface with verrucose bulges to intestinal folds when mature, two types of external hairs composed of strongly encrusted true hairs and smooth, slender short hairs, ellipsoid to bean-shaped, smaller ascospores (25–30.5 × 9–11 μm).

##### Holotype.

China • Fujian Province, Fuzhou City, Minhou County, Sandiejing National Forest Park, 26°26'N, 119°17'E, ca 550 m alt., on rotten fallen sticks or wood surrounded by mosses on riverside in moist forest mixed with evergreen broad-leaved trees and bamboo, 9 Nov. 2023, S. A. Chen (CSA–455, IBK, holotype!) (ITS: PQ489315; LSU: PQ187431; *rpb1*: PV247123; *rpb2*: PV296008; *tef1-α*: PV296003).

##### Description.

***Ascomata*** small to medium-sized, subsessile to distinctly stipitate. ***Apothecium*** cupulate, bowl-shaped to long funnel-shaped, up to 15–60 mm in diameter, 20–30 mm in high; margin entire and involute. ***Hymenium surface*** glabrous, initially without ornamentation or wrinkled, with verrucose bulges to intestinal folds at maturity, burlywood (3E8), yellowish-brown (4D8) to rosy brown (6E8), lighter in margin, color unchanging when bruised, darkening and cracked after drying. ***External surface*** velvety, densely dotted with droppable and fine grey (9D1) warts, with radial and forked ridges or crests not reaching margin, tapering towards the base or stipe, slate grey (12D1), dim grey (21D1) to off-black (4F8), covered by subiculum. ***Stipe* (when present)** up to 5 mm in diameter, 19 mm in high, uneven or foveolate, concolor with the external surface. ***Subiculum*** off-black (4F8), mussel base byssal-like, dense, soft, and fibrous, mixed or flexuous. ***Flesh*** thin, gelatinous, elastic, coco-nata white (1A1). ***Odor* and *taste*** not special. ***Anamorph*** not observed.

**Figure 13.**
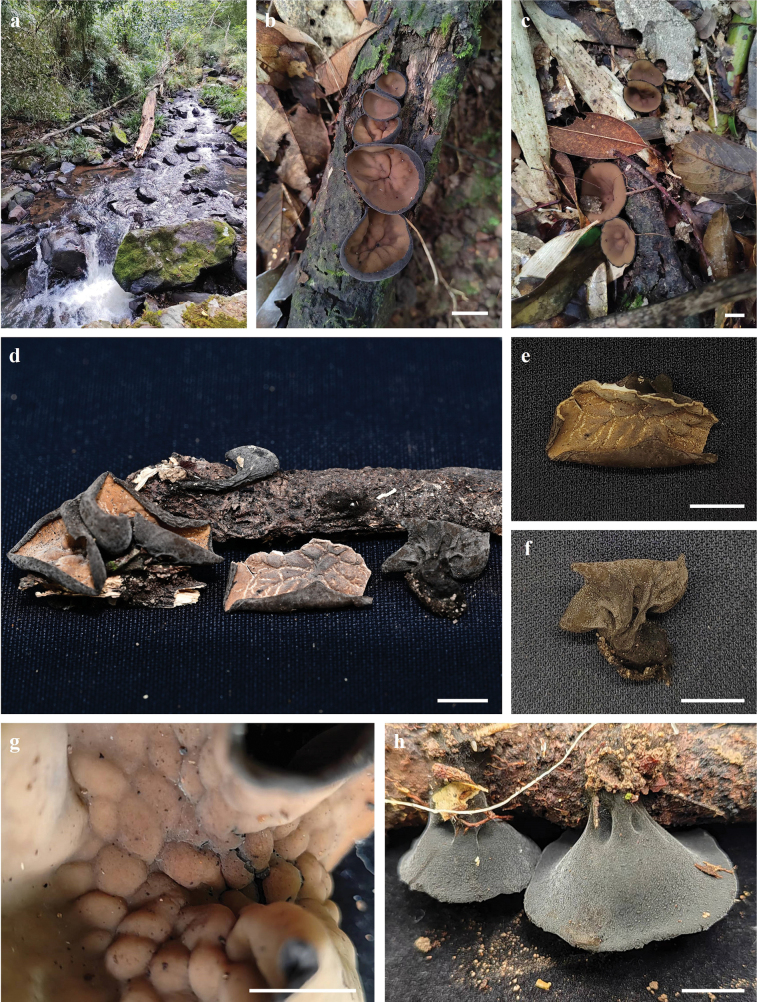
Ecological and macroscopic characteristics of *Urnula auricularioides*, photos by S. A. Chen; b, d, e, f, g. from holotype CSA-455 (IBK!); c, h. from CSA-454 (IBK!). a. Habitat where the specimen grew, a moist forest mixed with evergreen broad-leaved trees and bamboo on riverside; b, c. Fresh ascomata with a hymenium surface that is unfolded when immature and variously wrinkled when mature, growing on rotten branch surrounded by mosses; d, f. Dried materials; g. Hymenium surface with dense verrucose bulges; h. Velvety external surface of fresh specimens with greyish warts. Scale bars: 10 mm (b–h).

**Figure 14.**
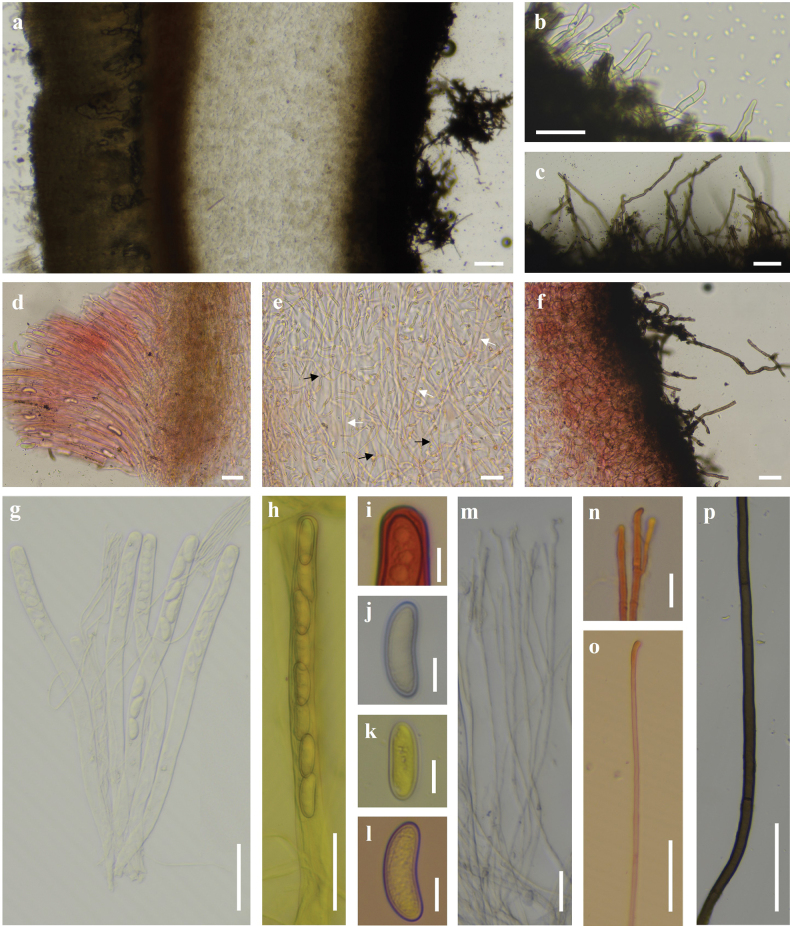
Microscopic structures of *Urnula auricularioides*, photos by S. A. Chen, from holotype CSA-455 (IBK!). a. Cross section through apothecia; b. Type 1 of external hairs; c. Type 2 of external hairs; d. Hymenium and subhymenium in Congo red; e. Medullary excipulum with two types of hyphae (type I indicated by black arrows; type II indicated by white arrows) in Congo red; f. Ectal excipulum and external hairs in Congo red; g. Asci with mature and immature ascospores; h. An ascus with 8 developed ascospores in Meltzer’s reagent; i. Apical apparatus of ascus in Congo red; j. Ascospore in distilled water; k. Ascospore in Meltzer’s reagent; l. Ascospore in Congo red; m. Straight to hooked hymenial hair and paraphyses; n. Tips of paraphyses in Congo red; o. Hymenial hair in Congo red; p. Hypha of subiculum. Scale bars: 100 μm (a); 50 μm (g, o, p); 20 μm (b, d–f); 10 μm (i–n).

**Figure 15.**
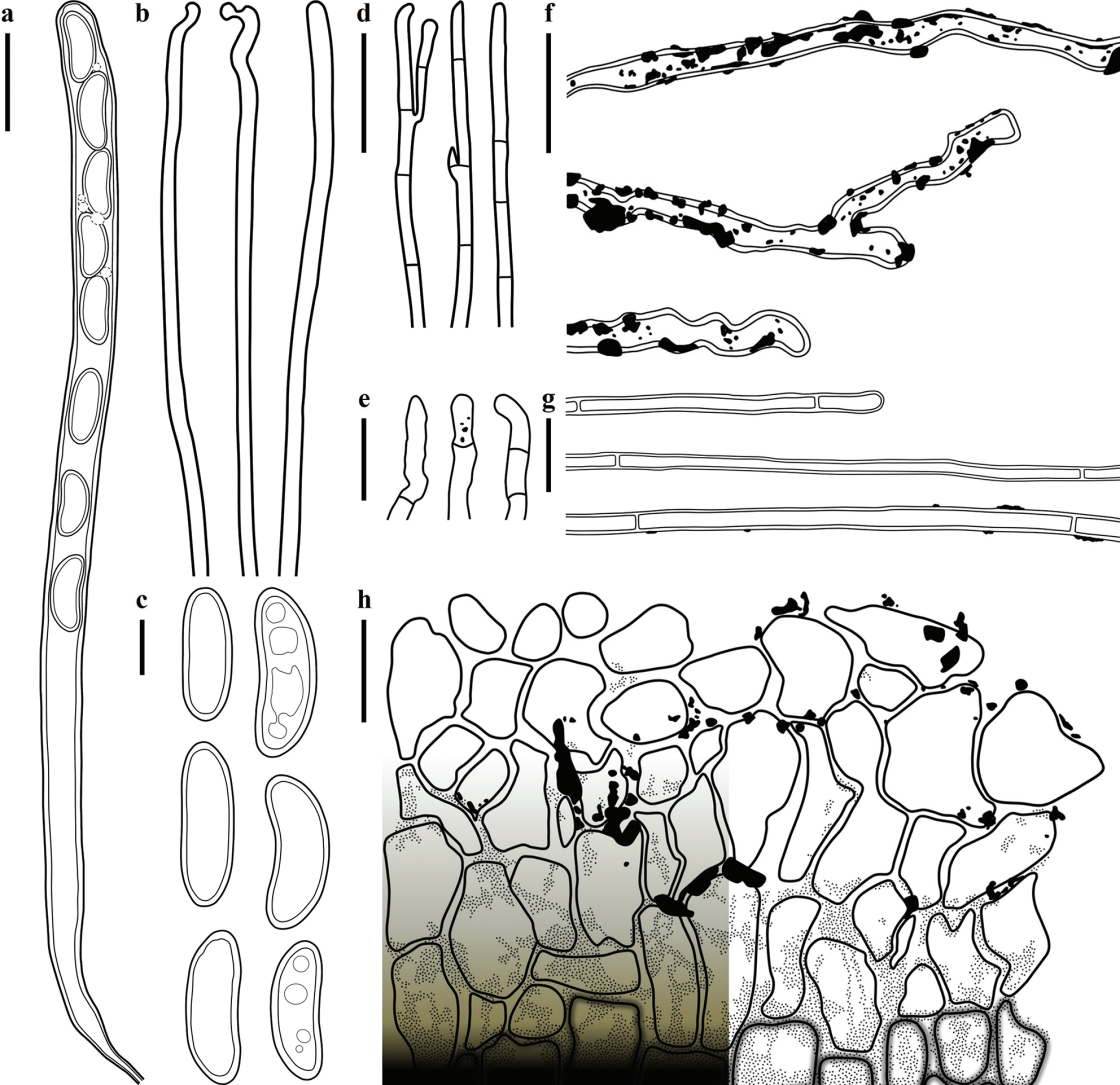
Microscopic structures of *Urnula auricularioides*, drawing by S. A. Chen, from holotype CSA-455 (IBK!). a. An ascus with 8 developed ascospores; b. Hymenium hairs; c. Ascospores; d. Paraphyses; e. Type 1 of external short hairs; f. Type 2 of external long hairs; g. Hypha of subiculum; h. A part of ectal excipulum. Scale bars: 50 μm (a); 20 μm (b, d, f, g, h; b, d sharing the same bars); 10 μm (c, e).

***External hairs*** mainly of two types, although intermediates forms may exist: 1) short, hyphoid, septate, cylindrical, slightly to distinctly curved or flexuous, subhyaline with a very pale yellow tint but occasionally dark brown (5E8) to dull black (18F8) for a granular intracellular pigment in part, thin-walled, 2–4 μm in diameter, subcylindrical, tips rounded to slightly subcapitate, surface smooth (Figs 14b, 15e); 2) long, true hairs, undulated, almost non-septate, some branched, tips rounded to truncated, surface unevenly covered by a granular to agglomerate encrustation, 4–7 μm in diameter, with moderately thick walls, up to 1 μm thick, light brown (4C7) or grayish olive (29D5) to dark brown (5E8) and off-black (4F8) (Figs 14c, 15f). ***Ectal excipulum*** of a *textura subglobulosa* to *textura angularis*, *textura irregularis* made up of properly thick-walled (up to 4 μm) cells up to 5–35 μm in length and/or width, tawny (4C5) to dark brown (5E8) and dull black (18F8), not or slightly encrusted, 95–240 μm thick (Fig. 14f). ***Medullary excipulum*** of a loose *textura intricata* immersed in a highly gelatinous matrix, approximately 260–510 μm thick (Figs 14e, 15h), mainly with two types of hyphae: I) short, septate, subhyaline or pale yellow (1A5), thin-walled, arboriform and flexuous, 2–3.5 μm in wide; II) long (relative to type I), septate, concolor with type I hyphae, thin-walled, cylindrical, almost no branched, roughly parallel to subhymenium, 1.5–3 (5) μm in wide. ***Subhymenium*** of a dense *textura intricata* of closely septate hyphae, surface smooth, up to 3 μm in diameter, thick-walled, tawny (4C5) to light brown (4C7), dark brown (5E8) at low magnification, 50–220 μm thick (Fig. 14d). ***Asci*** cylindrical, operculate, with a tapered or attenuated base, rounded at apex, 8-spored, 240–410 × 11–18 μm, non-amyloid (Figs 14g–i, 15a). ***Ascospores*** uniseriate, smooth, sometimes curved, thick-walled, rounded at both ends, heterogeneous in shapes depending on the spacial position and growth stage of the spores, pale yellow (1A5) or chartreuse (30A7) to grayish olive (29D5), incipiently ellipsoid to allantoid with walls up to 3 μm thick, becoming oblong to bean-shaped with walls 1–2.5 μm thick, [40/4/2] (23.5) 25–30.5 (32) × (8.5) 9–11 (11.5) μm, Q _values_ = (2.31) 2.46–3.1 (3.3), non-amyloid, with 0–5 lipid bodies (Figs 14i, k, l, 15c). ***Paraphyses*** filiform, not or slightly extending the length of asci, septate, cylindrical, straight to slightly curved, dark coffee brown (4E8) due to the extracellular amorphous pigments, darkening toward the upper part but deposited by dense off-black (18F8) pigments near the base, (1) 1.5–2.5 μm in diameter, tips rounded to finger-shaped, sometimes branched 1–3 times (Figs 14m, n, 15d). ***Hymenial hairs*** abundant, cylindrical, long as the paraphyses, non-septate, 2.5–3.5 (4.5) μm in diameter, concolor with the paraphyses due to the homogeneous pigments, agglutinating or intermingle with the paraphyses to bundles, tips rounded to slightly subcapitate, straight, curved, or flexuous to almost hooked (Figs 14o, 15b). ***Subiculum*** grayish olive (29D5), tawny (4C5), and rosy brown (6E8) to dark brown (5E8), septate, with a slightly thick wall, up to 1 μm thick, slightly curved, unbranched, not or slightly encrusted by an extracellular dark brown (5E8) to dull black (18F8) pigment, (2) 4.5–6 μm in diameter (Figs 14p, 15g).

**Figure 16.**
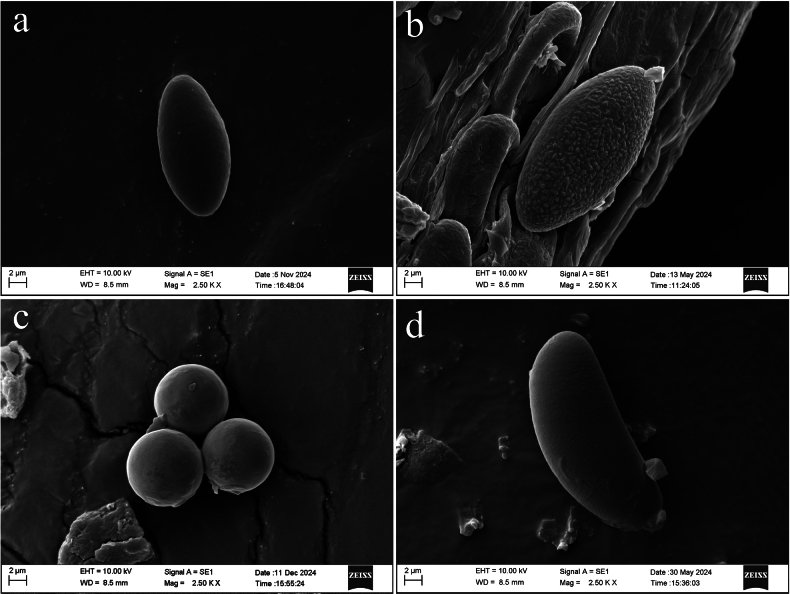
Scanning electron micrograph of basidiospores of the four new species of Sarcosomataceae from China (photos by J. R. Liu). a. *Plectania huapingensis*; b. *Plectania damingshanensis*; c. *Pseudoplectania aureonigrescens*; d. *Urnula auricularioides*. Scale bars: 2 µm (a–d).

##### Habitat.

Growing in gregarious groups on rotten fallen branches or twigs surrounded by mosses on riverside, half-buried in the litter layer in moist forest mixed with evergreen broad-leaved trees and bamboo.

##### Geographic distribution.

So far, only known from Fujian Province, China.

##### Other material examined.

China • Fujian Province, Fuzhou City, Minhou County, Sandiejing Forest Park, 26°26'N, 119°17'E, ca 550 m alt., 9 Nov. 2023, S. A. Chen, CSA-454 (IBK!) (ITS: PQ489472, LSU: PQ187433; *tef1-α*: PV296002).

##### Notes.

*Urnula auricularioides* is morphologically similar to *Urnula versiformis*, sharing most characteristics but distinctly differing from the latter by its slender external short hairs (2–4 μm in wide *vs.* 5–7 μm in wide) and small ascospores (25–30.5 × 9–11 μm *vs.* 30–35 × 10–12 μm) (Wang and Huang 2015). *U. auricularioides* is also similar to *U. campylospora* and *U. ailaoshanensis* in morphology. However, *U. campylospora* produces reticulate external surface and two-typed external hairs composed of heavily encrusted short hairs (5–6 μm in wide) and mainly smooth true hairs (Carbone and Agnello 2013), while *U. auricularioides* is evidently distinguished by its ridged external surface and smooth, slender external short hairs (2–4 μm in wide) and strongly encrusted true hairs; *U. ailaoshanensis* characteristically produces reticulate external surface and one-typed external hairs (Lu et al. 2023). In the phylogenetic analysis, *Urnula auricularioides* is relatively close to *Urnula himalayana*, but it can be easily separated on multiple morphological characteristics. *U. auricularioides* produces entire margin, smooth and slender external short hairs (2–4 μm in wide *vs.* 6–7 μm in wide), and smaller ascospores (25–30.5 × 9–11 μm *vs.* 24–33.6 × 10–13.8 μm). Additionally, the external surface appearance of both species is also clearly different. *U. himalayana* has a reticulate pattern without warts formed by vertically joined folds or veins (Wang et al. 2018), whereas *U. auricularioides* possesses a ridged surface covered with vertical folds or crests with visible warts.

### ﻿Key to accepted species of *Urnula*

*Urnula* species lacking molecular data or a clear original diagnosis are not included (viz., *Urnula brachysperma*, *U. groenlandica*, *U. mexicana*, *U. microcrater*, and *U. viridirubescens*).

**Table:** 

1	Mature ascospores ellipsoid to allantoid, bean-shaped or reniform-like	**2**
–	Mature ascospores ellipsoid to short clavate	**6**
2	External surface velvety to warty	**3**
–	External surface velvety without warts	**5**
3	External short hairs mostly smooth, slender	***Urnula auricularioides***
–	External short hairs encrusted to heavily encrusted, thick	**4**
4	Ectal excipulum 40–60 μm thick; base vertically corrugated; mature ascospores 22.7–31.8 × 9.1–13.6 μm	***U. campylospora***
–	Ectal excipulum 50–125 μm thick; base ridged; mature ascospores 26–32 × 9.5–11.5 μm	***U. versiformis***
5	Two-typed external hairs	***U. himalayana***
–	One-typed external hairs	***U. ailaoshanensis***
6	External surface with warts or tubercles	**7**
–	External surface with patches of issue or tomentum	**8**
7	Ascomata with a well-developed stipe; mature ascospores thick-walled; ectal excipulum with crystals	***U. mediterranea***
–	Ascomata with a short to well-developed stipe; mature ascospores thin-walled; ectal excipulum without crystals	***U. hiemalis***
8	Ascomata sessile; external surface with patches of grey tomentum within the large radial folds	***U. padeniana***
–	Ascomata subsessile to stipitate; external surface with yellow to brown patches of issue	**9**
9	Asci short; paraphyses slender	***U. craterium* complex**
–	Asci obviously long; paraphyses thick	***U. subcrateria***

## ﻿Discussion

As a cosmopolitan family, Sarcosomataceae has been explored for many decades, representing an interesting group of fungal taxa capable of growing in tropical, subtropical, temperate, and even cold climates (Carbone et al. 2013a, 2014a; Blanchette et al. 2016; Van Vooren and Mauruc 2020). Previous studies of sarcosomataceous species were mainly confined to temperate regions (Dissing 1981; Calonge et al. 2003; Carbone et al. 2013a, 2014a, 2015a; Glejdura et al. 2015; Pfister and LoBuglio 2018; Saitta 2020; Popov and Carbone 2021; Carbone and Rocha 2024). The current study lays emphasis on updating the diversity, taxonomy, and delimitation of sarcosomataceous fungi in subtropical China, based on ecological observations, morphological characteristics, and multi-gene phylogenetic analysis. Four new species were scientifically verified, namely *Plectania damingshanensis*, *P. huapingensis*, *Pseudoplectania aureonigrescens*, and *Urnula auricularioides*. In addition, two taxa with new distribution records were revealed: *Plectania lutea* and *Urnula ailaoshanensis*. An updated list of Sarcosomataceae species in China is shown in Table 2, in which a total of 25 species were recorded in the subtropical region, except for *Urnula craterium*, indicating the extremely rich diversity of these ascomycetes in subtropical China.

**Table 2.** List of Sarcosomataceae taxa reported from China. A tick after taxa indicates that its distribution in China is supported by molecular data. Location of taxa is based on relevant reports with or without molecular data. The letters STR, TE, and TR in climate are indicated as subtropical, temperate, and tropical, respectively.

Genus	Taxa	Location	Climate	References
*Donadinia* (3)	*D. echinacea* √	Yunnan	STR	Zeng et al. (2021)
	D. cf. helvelloides	Tibet (Nyingchi)	STR	Zhuang (2004)
	*D. nigrella*	Tibet (Nyingchi)	STR	Xu (2000); Zhuang (2004)
*Galiella* (2)	*G. amurensis* √	Heilongjiang, Jilin, Sichuan, Tibet (Nyingchi)	STR, TE	Zhuang (2004); Popov and Carbone (2021)
	*G. sinensis*	Fujian, Yunnan	STR	Cao et al. (1992); Zhuang (2004)
*Plectania* (10)	*P. damingshanensis* √	Guangxi	STR	This study
	*P. huapingensis* √	Guangxi	STR	This study
	*P. lutea* √	Guangxi	STR	Mou and Bau (2021)
	*P. melastoma*	Tibet (Nyingchi)	STR	Xu (2000)
	*P. nigrella*	Tibet (Nyingchi)	STR	Xu (2000)
	*P. platensis*	Hubei	STR	Xu (2000); Zhuang (2004)
	*P. rhytidia*	Tibet (Nyingchi)	STR	Xu (2000); Zhuang (2004)
	*P. sichuanensis* √	Sichuan	STR	Zeng et al. (2021)
	*P. submilleri* √	Yunnan	STR	Hyde et al. (2024)
	*P. yunnanensis*	Yunnan	STR	Zhuang and Wang (1998); Zhuang (2004)
*Pseudoplectania* (5)	*Ps. aureonigrescens* √	Fujian	STR	This study
	*Ps. globospora* √	Yunan	STR	Zeng et al. (2024)
	*Ps. mystica* √	Zhejiang	STR	Lin et al. (2024)
	*Ps. nigrella* √	Heilongjiang, Hubei, Sichuan, Tibet (Nyingchi)	STR, TE	Xu (2000); Zhuang (2004); Lin et al. (2024)
	*P.. sinica* √	Fujian	STR	Zhang and Zhang (2020)
*Urnula* (6)	*U. auricularioides* √	Fujian	STR	This study
	*U. ailaoshanensis* √	Guangxi, Yunnan	STR	Lu et al. (2023); This study
	*U. campylospora*	Guangxi, Hainan, Yunnan	STR, TR	Zhuang (2004)
	*U. craterium*	Heilongjiang	TE	Zhuang and Wang (1998); Zhuang (2004)
	*U. subcrateria* √	Yunnan	STR	Lu et al. (2023)
	*U. versiformis* √	Taiwan	STR	Wang and Huang (2015)

Previous studies related to the classification of Sarcosomataceae only performed one- or two-locus phylogenetic analyses. In the phylogenetic analysis using ITS and nrLSU sequences by Carbone et al. (2013a), the results revealed that six monophyletic clades corresponded well to the six accepted genera: *Donadinia*, *Galiella*, *Plectania*, *Pseudoplectania*, *Sarcosoma* and *Urnula*. However, judging from our phylogenetic result, *Donadinia*, *Pseudoplectania*, *Sarcosoma*, and *Urnula* appeared to be monophyletic with significant support, while *Galiella* and *Plectania* did not form two distinct monophyletic groups but instead composed a single complex: the *Plectania* s. lat. clade. Moreover, with wider taxon sampling, Zeng et al. (2021) also proposed a similar division based on an ITS + nrLSU dataset including 126 Sarcosomataceae taxa. However, upon further comparison with our topology, there are some differences in the division of *Galiella* and *Plectania*. In their study, *Plectania* was divided into three divergent clades (Plectania I–III), and *Galiella* was represented only by *G. rufa*, grouped as sister to *Plectania* clade I. In our study, the *Plectania* s. lat. clade is composed of several lineages, including *Galiella*, which is represented by two species (*G. amurensis* and *G. rufa*). With the application of more extensive shallow sequencing data, our study revealed inconsistency in the divisions between our multi-locus phylogenetic analysis and the two comparative cases. This suggests that ITS-based or ITS + nrLSU-based analyses, when based on limited specimen sampling, are insufficient to investigate the relationship between *Galiella* and *Plectania*.

As mentioned, the *Plectania* s. lat. clade encompasses both *Galiella* and *Plectania*. Species within this complex group exhibit a wide range of morphological variation, offering several interspecific distinguishing features. The main diagnostic characteristics for differentiating *Plectania* species include apothecium size, stipe length, color of the hymenium surface, density of tomentum on the external surface, presence of one or two types of external hairs, and ascospore size and ornamentation.

Two new members were collected from Guangxi: *Plectania damingshanensis* and *P. huapingensis*. The former is distinguished by cupulate ascomata, a brownish-orange to light-brown hymenium surface, a velvety and ridged external surface, and elliptical ascospores. *Plectania huapingensis* is recognized by disc-shaped or irregularly margined disc-shaped ascomata, an external surface adorned with brown-orange to brownish-red particles, and smooth elliptical ascospores. The macroscopic and microscopic structures of the two species are similar to those of *Galiella* species in possessing gelatinous flesh and a bright hymenium surface. Further clarification requires more refined morphological analysis and accurate molecular systematic studies.

Additionally, on intergeneric aspects, the delimitation between *Galiella* and *Plectania* lacks clarity. In existing research, species with *Plectania*-like and dark-colored apothecia, thin medullary excipulum, and ellipsoid ascospores were placed in *Plectania*, while *Sarcosoma*-like and bright-colored apothecia, thick medullary excipulum, and verrucose ellipsoid ascospores were placed in *Galiella* (Korf 1957a). Recently, gelatinous apothecia, verrucose ascospores, and smooth external hairs were recognized as the main distinguishing features of the genus *Galiella* (Carbone et al. 2015b). Exceptionally, *P. damingshanensis* and *P. lutea* blur these criteria, as they possess gelatinous apothecia; yellow, light brown, or orange-brown hymenium surfaces; and verrucose or vermicular ellipsoid ascospores with smooth external hairs. Notably, *P. damingshanensis* also has a thick and watery medullary excipulum.

From a phylogenetic point of view, *Galiella* and *Plectania* species cluster into a monophyletic group with strong support (MLB = 78%, BPP = 1). Moreover, the two *Galiella* lineages—*G. amurensis* and *G. rufa*—did not even cluster together with strong support. Based on both morphological characters and topological evidence, this seems to indicate that there is no obvious heterogeneity at the genus level between *Galiella* and *Plectania*. However, we adopt a cautious position regarding this intricate group until more specimens are available and more convincing morphological criteria are proposed.

In *Pseudoplectania*, species within this monophyletic genus share highly similar macroscopic features and are recognized by tiny to medium-sized ascomata and globose to subglobose ascospores. The diagnostic characters, combining macro- and micro-morphological features, chiefly concern apothecium size, whether sessile or stipitate; paraphyses with or without hooked tips; one- or two-typed and coiled or straight external hairs; and ascospores with or without ornamentation and the position of the gelatinous sheath. Two other potentially useful characteristics are the presence or absence of the gelatinous sheath and the presence of large or small crystals in the hymenium, though these should be considered in conjunction with the freshness of collections.

Subclade I is represented only by the *Ps. melaena* complex, showing distinctly stipitate fruitbodies, paraphyses with often hooked tips, straight to slightly curved external hairs (but not coiled), and ascospores with an eccentrically positioned gelatinous sheath (Van Vooren et al. 2013). Within subclade II, *P.. episphagnum* and the *Ps. nigrella* complex (including *Ps. lignicola*) form a phylogenetically affinitive group. Their teleomorphs commonly share the characteristics of sessile to shortly stipitate fruitbodies, paraphyses tips not hooked, the presence of coiled external hairs, and ascospores with or without eccentrically to centrally arranged gelatinous sheaths, except for *P.. sinica* (Carbone and Agnello 2012; Carbone et al. 2013a, 2014a). Subclade III is composed of three identified species—*Ps. affinis*, *Ps. aureonigrescens*, and *Ps. globospora*—which possess sessile fruitbodies, paraphyses tips not hooked, straight to curved or flexuous external hairs (but not coiled), and smooth ascospores without any surrounding gelatinous sheath (Carbone et al. 2014a; Zeng et al. 2024).

Among these, *Ps. aureonigrescens*, collected from Fujian, differs from the other two related species by its bowl-shaped, petal-shaped to irregular apothecium; dull hymenium surface when mature; one-typed external hair, straight to distinctly curved or flexuous (not coiled); and the presence of crystals in the hymenium. Subclade IV comprises *Ps. africana*, *Ps. ericae*, *Ps. mystica*, and *P.. tasmanica*, which feature sessile to shortly stipitate fruitbodies, paraphyses tips not hooked, straight to curved or flexuous external hairs (but not coiled), and ascospores with an eccentrically arranged gelatinous sheath (Van Vooren et al. 2013; Carbone et al. 2014a; Carbone and Rocha 2024; Lin et al. 2024).

In *Urnula*, species are distinctive in their dark, bowl-shaped to funnel-shaped ascomata and smooth, elliptical to allantoid ascospores when mature. Among these species, the dominant diagnostic characters are stipe length; the appearance of the external surface or base; the presence of one or two types of external hairs; and the shape of the spores. The collection YC2023090928 (IBK), collected from Guangxi, belongs to *Urnula*. Unfortunately, we did not find any visible ascospores due to the immaturity of the material.

Within subclade A, *U. auricularioides*, collected from Fujian, is characterized by a velvety to warty external surface with ridges and a hymenium surface with verrucose bulges to intestinal folds when mature; two types of external hairs composed of strongly encrusted true hairs and smooth, slender, short hairs; and ellipsoid to bean-shaped ascospores. In addition, subclade A is seemingly confined to the Oceania–Asian distribution, sharing the characteristics of ellipsoid to allantoid, bean-shaped or reniform-like ascospores and a velvety to warty, ridged to corrugated external surface. According to the concept proposed by Korf (1957a), the species within the monophyletic subclade A are compatible with the morphology of Plectania sect. Curvatisporae. Here, it is worth mentioning that the revision of the type of *U. mexicana* (Ellis & Holw.) M. Carbone, Agnello, A.D. Parker & P. Alvarado revealed bean-shaped ascospores and one-typed external hairs, and it is so far confined to Mexico (Carbone et al. 2013c), showing morphological similarity to the species of subclade A. However, there is currently no sequence available for *U. mexicana* to confirm its exact phylogenetic position. Molecular data from specimens collected at the type locality are still needed.

Based on the analysis of morphological characters combined with topological structure, it can be hypothesized that two evolutionary directions of spore shape exist in Sarcosomataceae: from ellipsoid to spherical and from ellipsoid to allantoid. Ellipsoid spores are present in *Donadinia*, *Plectania* s. lat., and *Sarcosoma*. Conversely, as a sister clade to *Sarcosoma*, *Pseudoplectania* displays globose to subglobose spores. *Urnula* exhibits a mixture of ellipsoid and allantoid spores, with all *Urnula* species bearing allantoid spores clustered in subclade A. This study also suggests a trend of spore evolution from smooth to ornamented surfaces. As shown by icons in our topology (Fig. 1), species of *Pseudoplectania*, *Sarcosoma*, and *Urnula* uniformly possess smooth spores, whereas species of *Donadinia* are characterized by verrucose spores. Especially in *Plectania* s. lat., spore ornamentation patterns are variable—smooth, finely to obviously verrucose or vermicular, and grooved. Notably, spore surface area is influenced by both shape and ornamentation. These findings indicate two divergent trends in surface area: (1) spores evolving from ellipsoid to allantoid and smooth to ornamented, corresponding with an increase in surface area variation; and (2) spores evolving from ellipsoid to spherical, associated with a decline in surface area variation. These trends may reflect different life strategies. A larger surface area may enhance dispersal and colonization potential (Jacobsen et al. 2017; Maurice et al. 2021), whereas a smaller surface area may strengthen water retention capacity (Kauserud et al. 2008; Fan et al. 2023). Moreover, species with sheath-covered spores appear to be confined to *Plectania* s. lat. and *Pseudoplectania*, according to our topology. The function of the gelatinous sheath remains poorly understood, but it is probably associated with the protection of developing spores and the liberation of mature spores (Ruddick and Williams 1972; Williams et al. 1972).

In conclusion, this study concentrates on updating the diversity and taxonomy of Sarcosomataceae species in subtropical China. Combined with morphological examination and multi-locus phylogenetic analysis, four new species and two specimens with new distribution records were revealed and described, and keys for elucidating interspecific differences were provided. Moreover, we encourage further systematic studies with the aim of clarifying phylogenetic relationships within *Plectania* s. lat. and providing new insights into reliable intergeneric criteria to support a more natural classification and broader concepts within Sarcosomataceae.

## Supplementary Material

XML Treatment for Plectania damingshanensis

XML Treatment for Plectania huapingensis

XML Treatment for Plectania lutea

XML Treatment for Pseudoplectania aureonigrescens

XML Treatment for Urnula ailaoshanensis

XML Treatment for Urnula auricularioides

## ﻿Additional information

### Use of AI

No use of AI was reported.

### Author ORCIDs

Jin Rong Liu https://orcid.org/0000-0003-0843-2097

Deng Li https://orcid.org/0009-0009-9357-3214

Si Ang Chen https://orcid.org/0009-0007-7315-8107

Yan Cheng Zhang https://orcid.org/0009-0000-5278-1134

Guang Fu Mou https://orcid.org/0009-0007-2318-7807

Yan Liu https://orcid.org/0000-0001-8931-9656

Guang Rong Zhou https://orcid.org/0009-0001-6557-7092

Zhou Rong Tan https://orcid.org/0009-0000-1160-7041

Jian Hua Zhang https://orcid.org/0009-0007-9717-4996

### Data availability

All of the data that support the findings of this study are available in the main text.
